# Supplementary material for: Design, Synthesis, and Bioactivities of Novel Tryptophan Derivatives Containing 2,5-Diketopiperazine and Acyl Hydrazine Moieties
Source: Molecules. 2022 Sep 6;27(18):5758. doi: 10.3390/molecules27185758 (PMC9506431; doi:10.3390/molecules27185758)
Supplement: Supplementary file 1 [file molecules-27-05758-s001.zip › molecules-1896560-SI.pdf]

Supplementary Material

# Design, Synthesis, and Bioactivities of Novel Tryptophan Derivatives Containing 2,5-Diketopiperazine and Acyl Hydrazine Moieties

**Lili Li [1], Rongxin Yang [1], Jianhua Liu [1], Jingjing Zhang [1,2,*], Hongjian Song [1,*], Yuxiu Liu [1] and Qingmin Wang [1,*]**

[1] China State Key Laboratory of Elemento-Organic Chemistry, College of Chemistry, Frontiers Science Center for New Organic Matter, Nankai University, Tianjin 300071, China

[2] College of Basic Science, Tianjin Agricultural University, Tianjin 300384, China

* Correspondence: jingjingzhang216@outlook.com (J.Z.); songhongjian@nankai.edu.cn (H.S.); wangqm@nankai.edu.cn (Q.W.); Tel./Fax: +86-22-235-039-52 (Q.W.)

## Section S1: Copies of NMR spectra

**1**

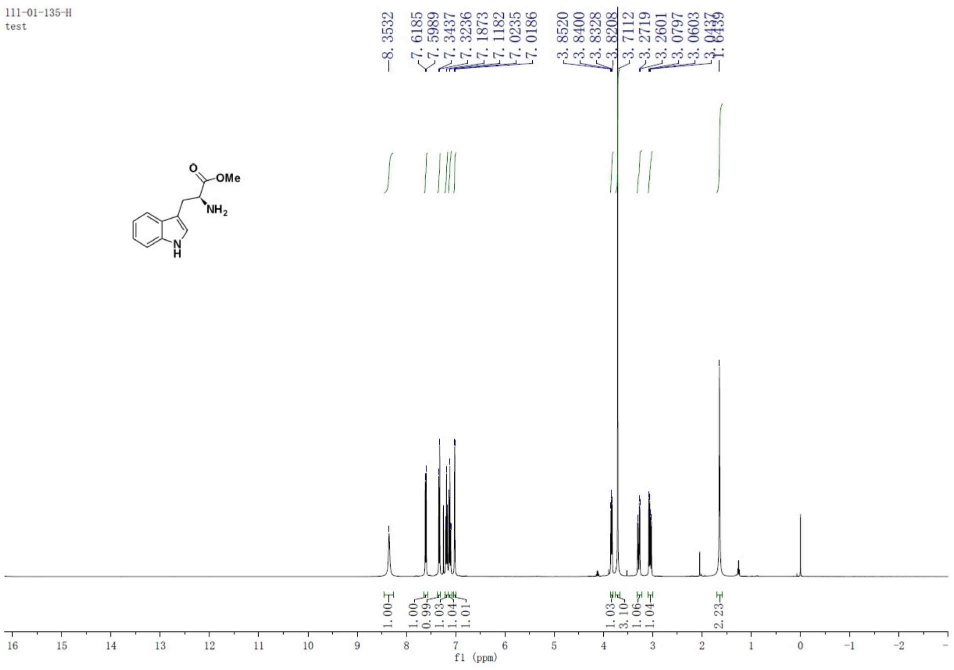


**Figure S1** $^{1}H$ NMR (400 MHz, $CDCl_3$) of **1**

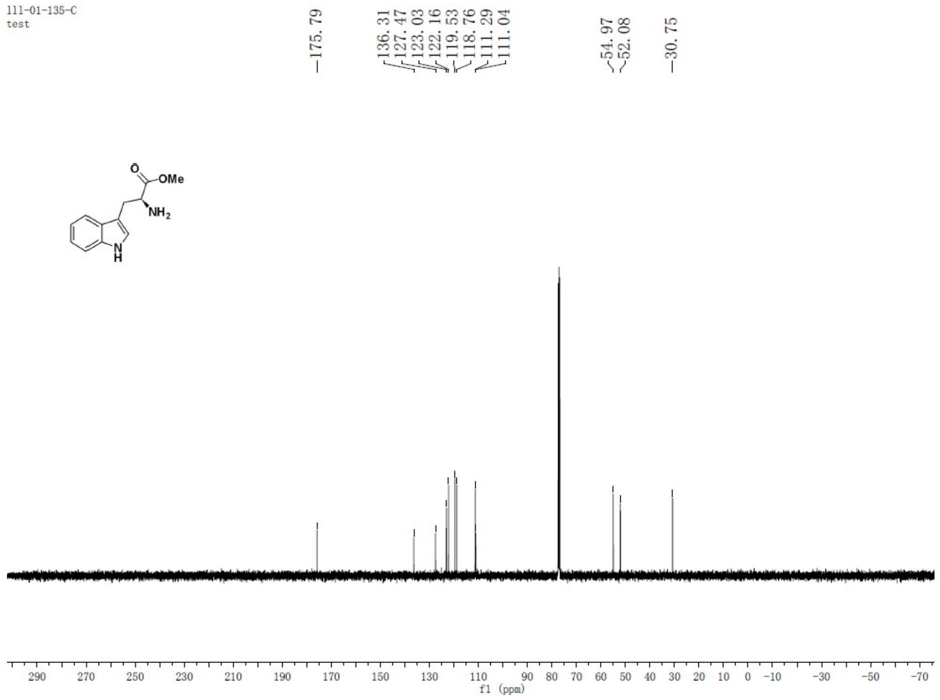


**Figure S2** $^{13}C$ NMR (100 MHz, $CDCl_3$) of **1**

**2**

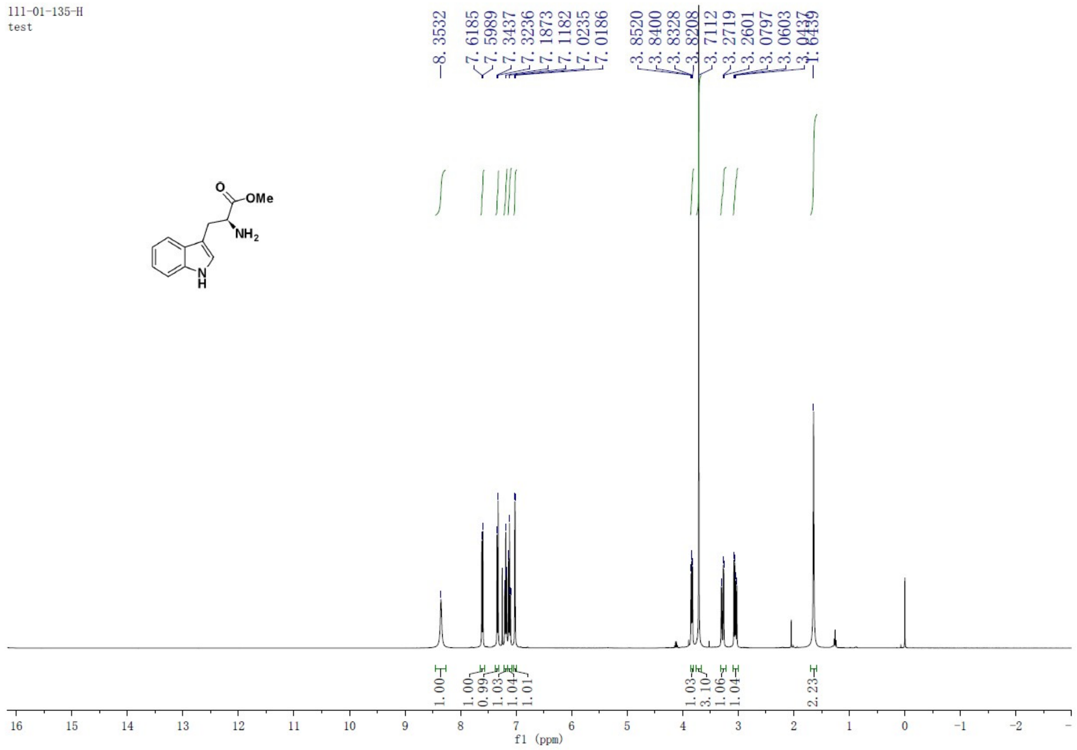


**Figure S3** $^{1}$H NMR (400 MHz, $CDCl_3$) of **2**

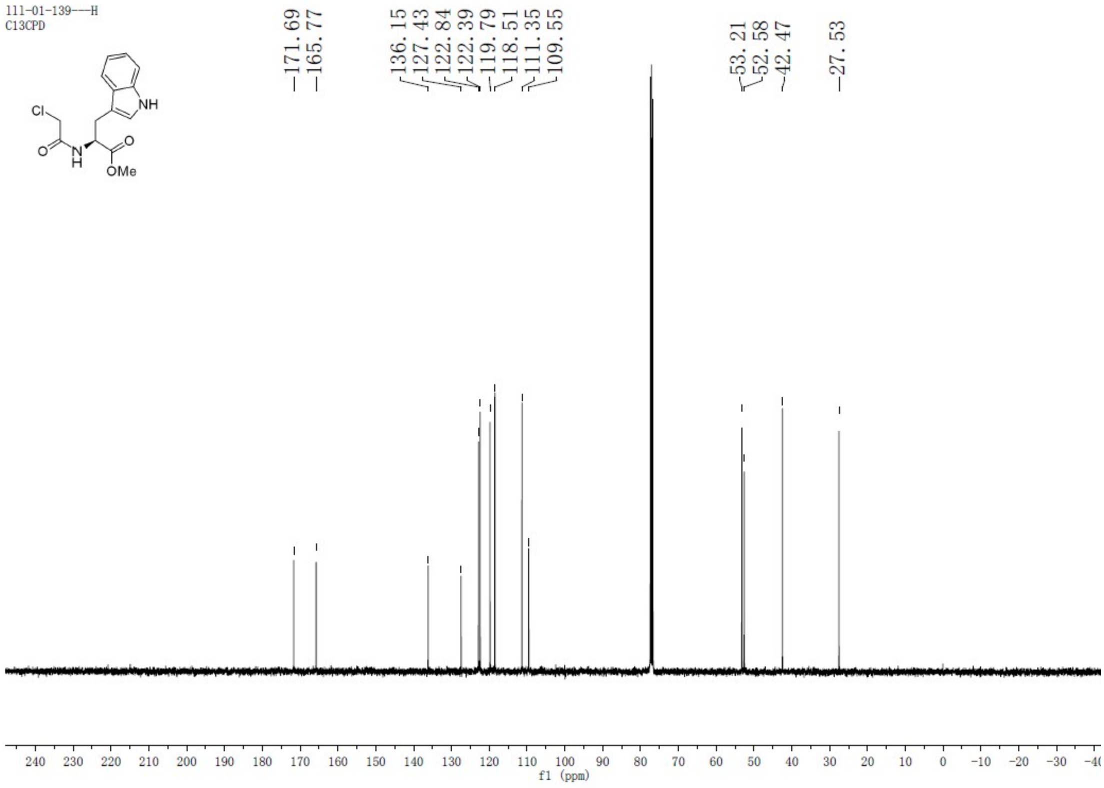


**Figure S4** $^{13}$C NMR (100 MHz, $CDCl_3$) of **2**

**3**

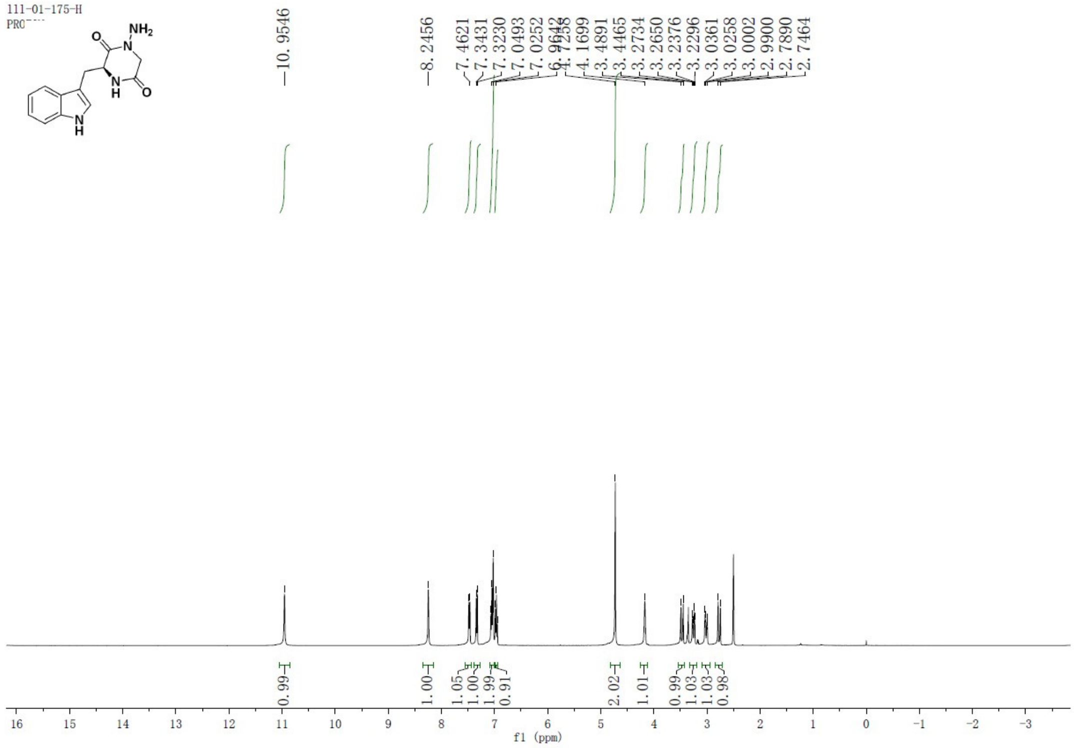


**Figure S5** $^{1}$H NMR (400 MHz, DMSO-$d_6$) of **3**

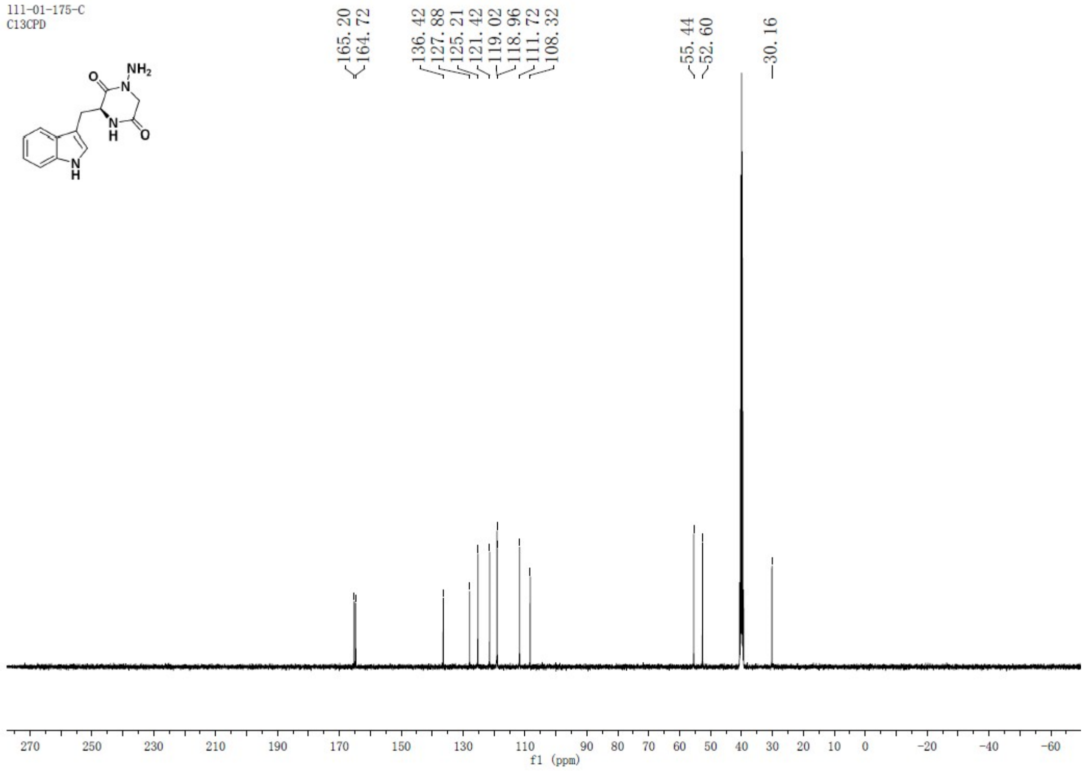


**Figure S6** $^{13}$C NMR (100 MHz, DMSO-$d_6$) of **3**

**4**

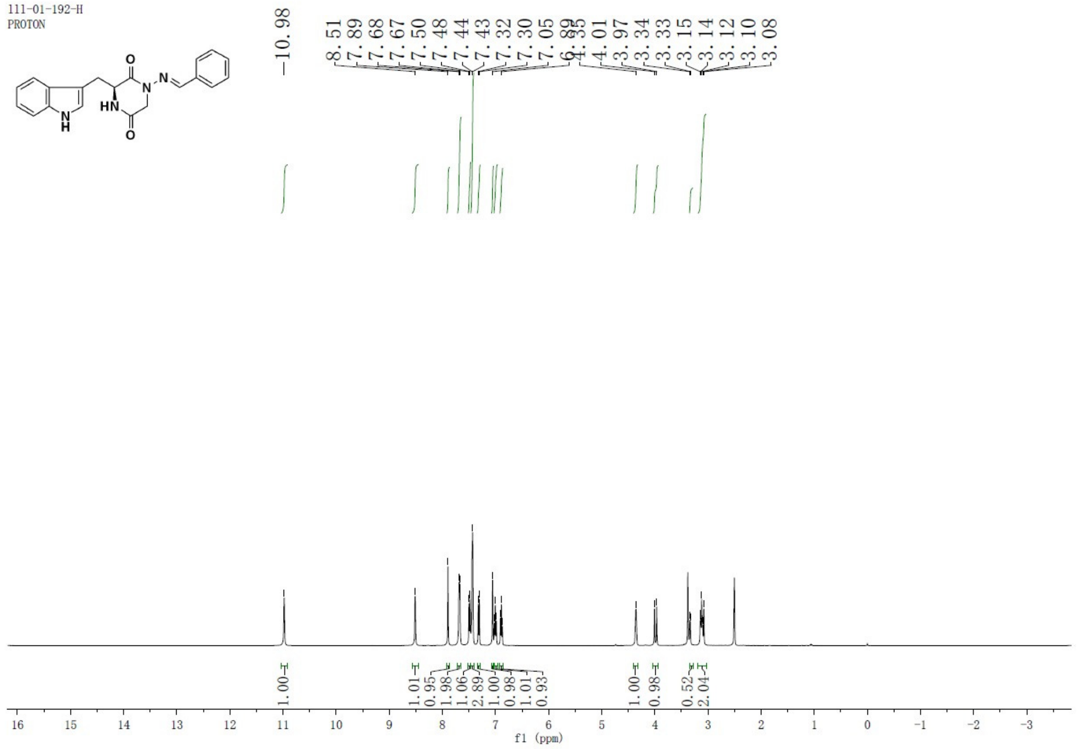


**Figure S7** $^{1}$H NMR (400 MHz, DMSO-$d_6$) of **4**

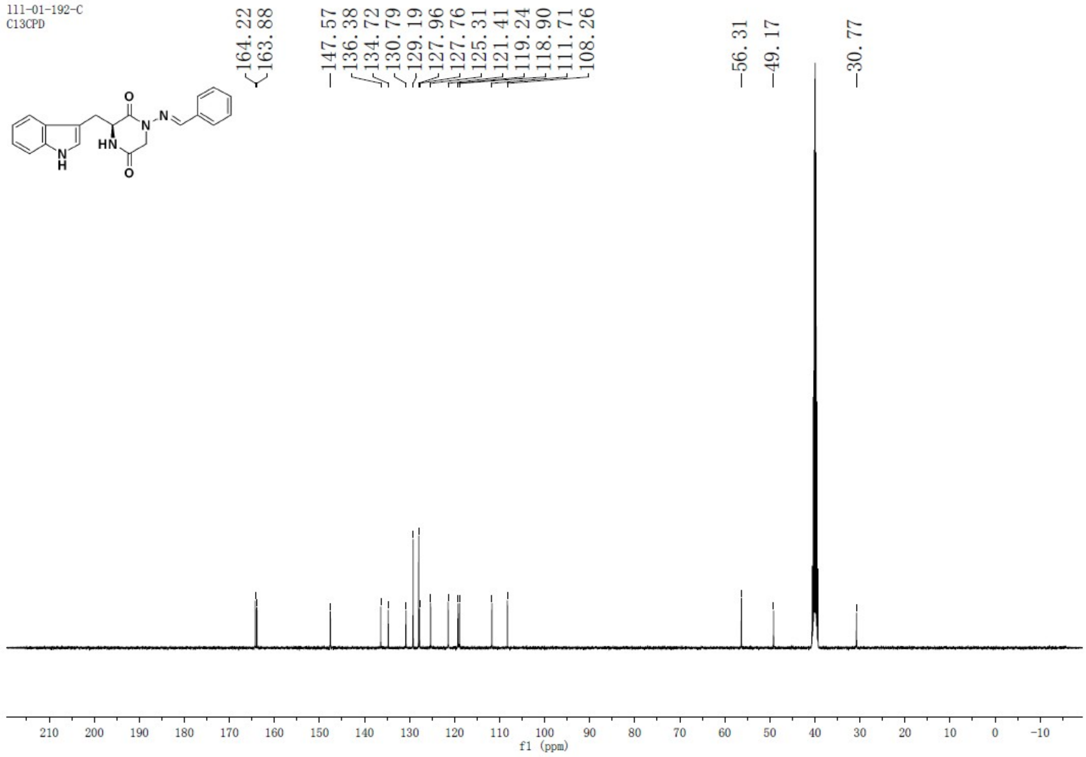


**Figure S8** $^{13}$C NMR (100 MHz, DMSO-$d_6$) of **4**

# 5

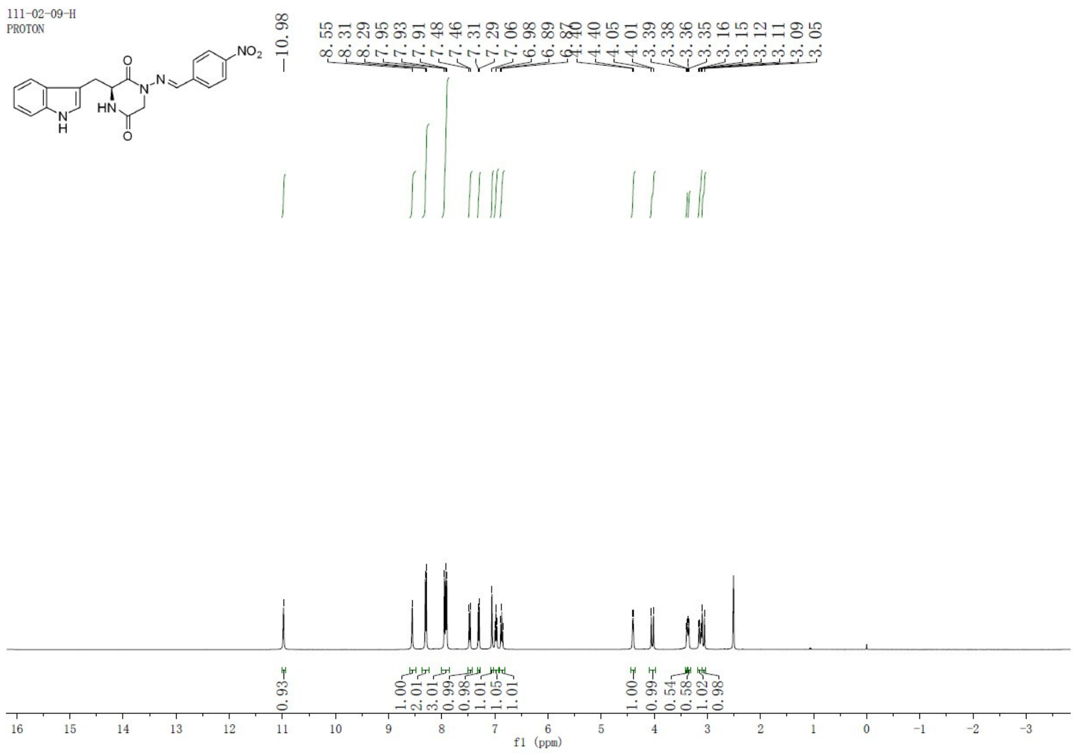


**Figure S9** $^{1}$H NMR (400 MHz, DMSO-$d_6$) of **5**

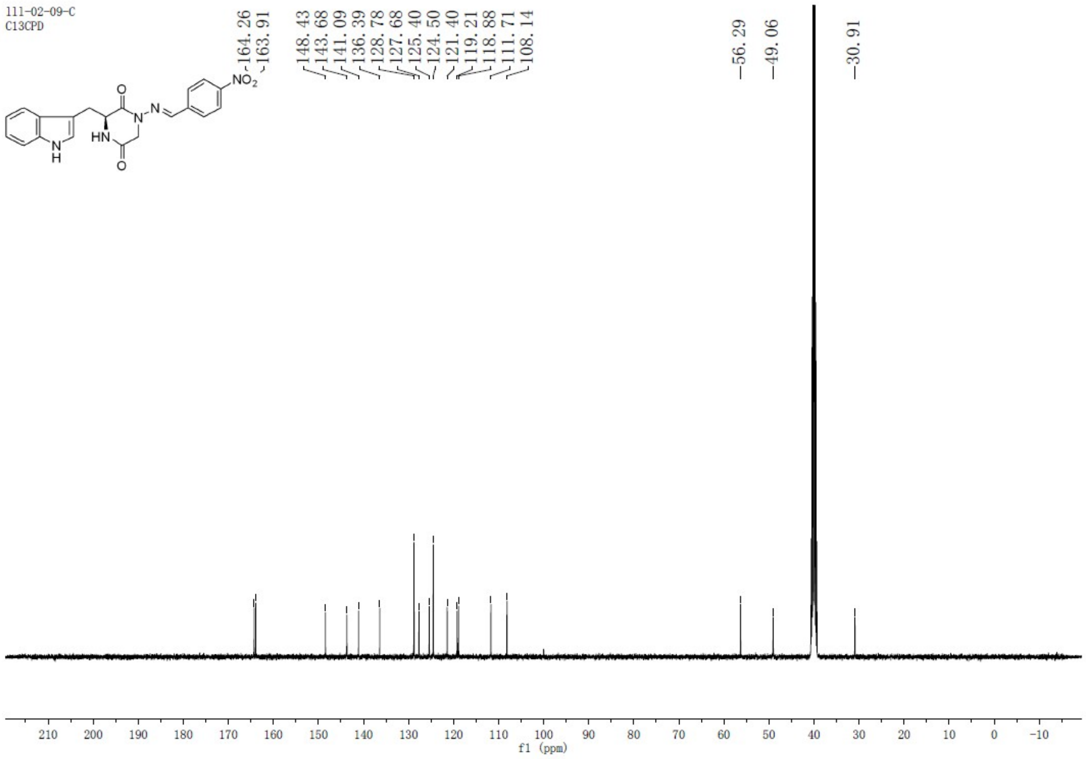


**Figure S10** $^{13}$C NMR (100 MHz, DMSO-$d_6$) of **5**

**6**

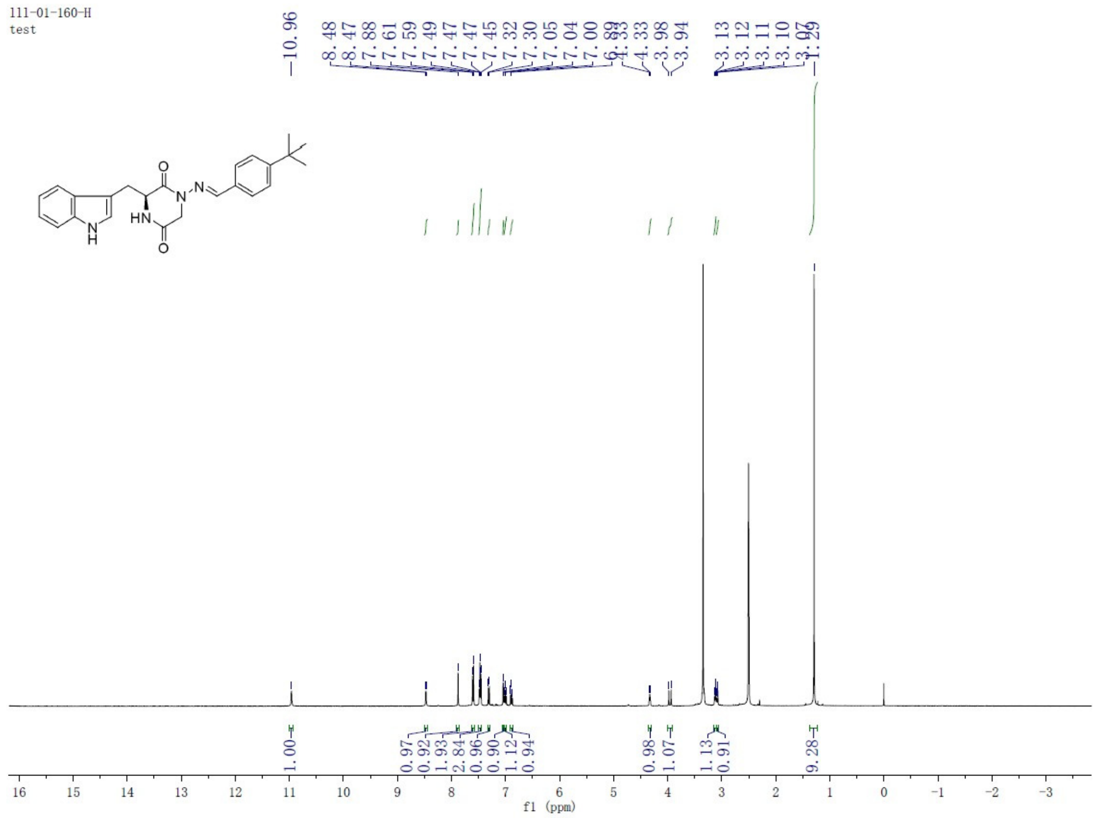


**Figure S11** $^{1}$H NMR (400 MHz, DMSO-$d_6$) of **6**

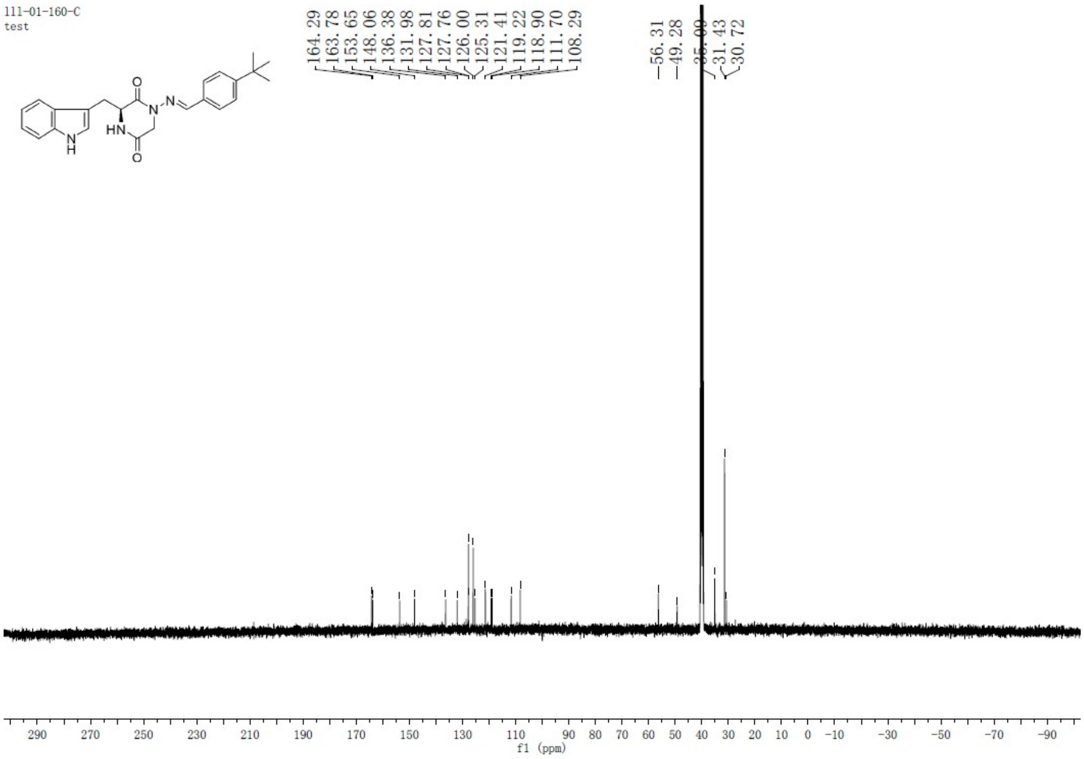


**Figure S12** $^{13}$C NMR (100 MHz, DMSO-$d_6$) of **6**

# 7

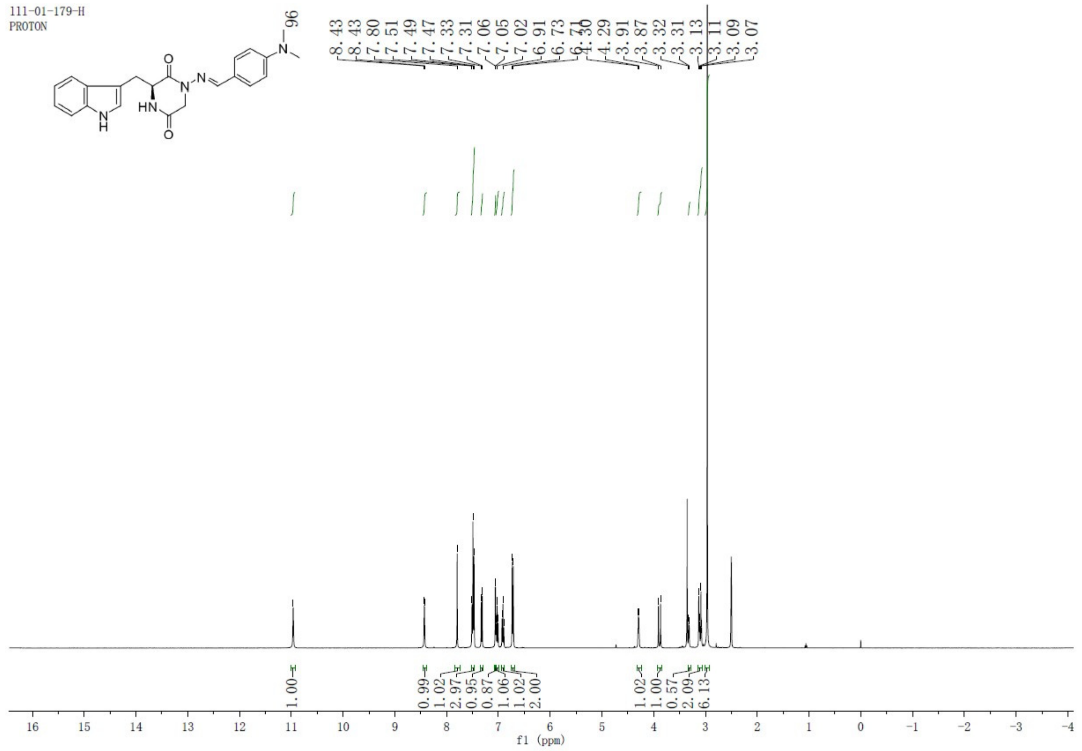


**Figure S13** $^{1}$H NMR (400 MHz, DMSO-$d_6$) of **7**

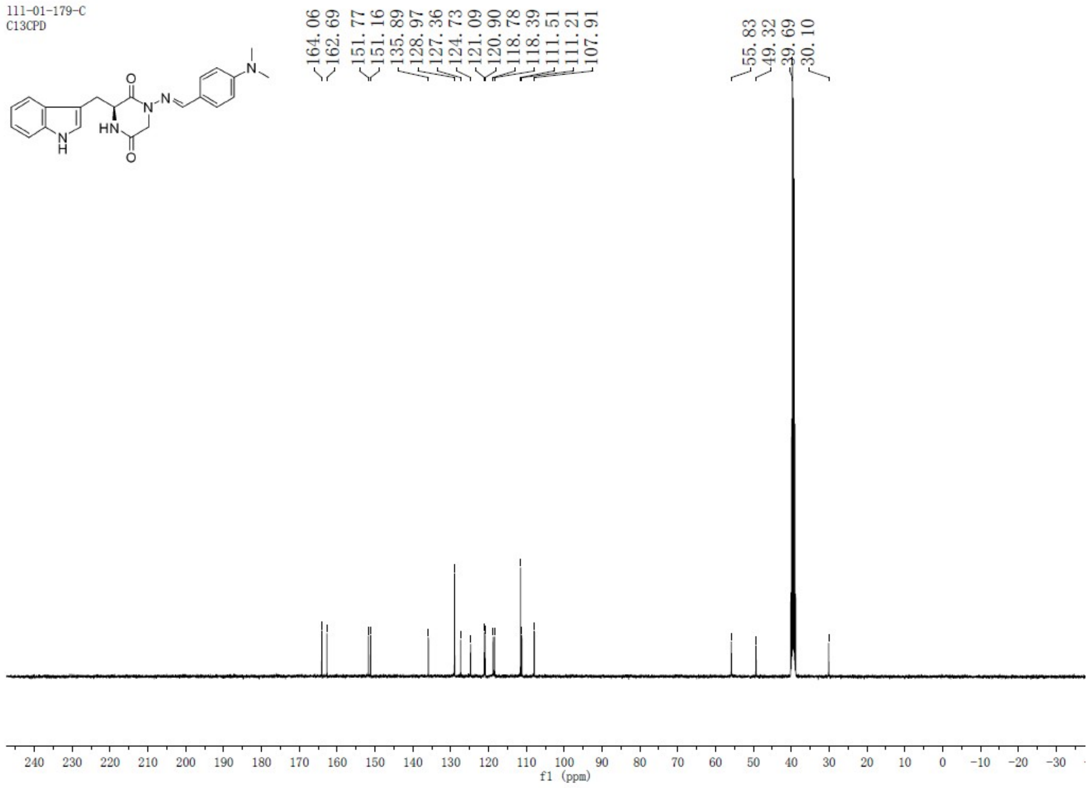


**Figure S14** $^{13}$C NMR (100 MHz, DMSO-$d_6$) of **7**

**8**

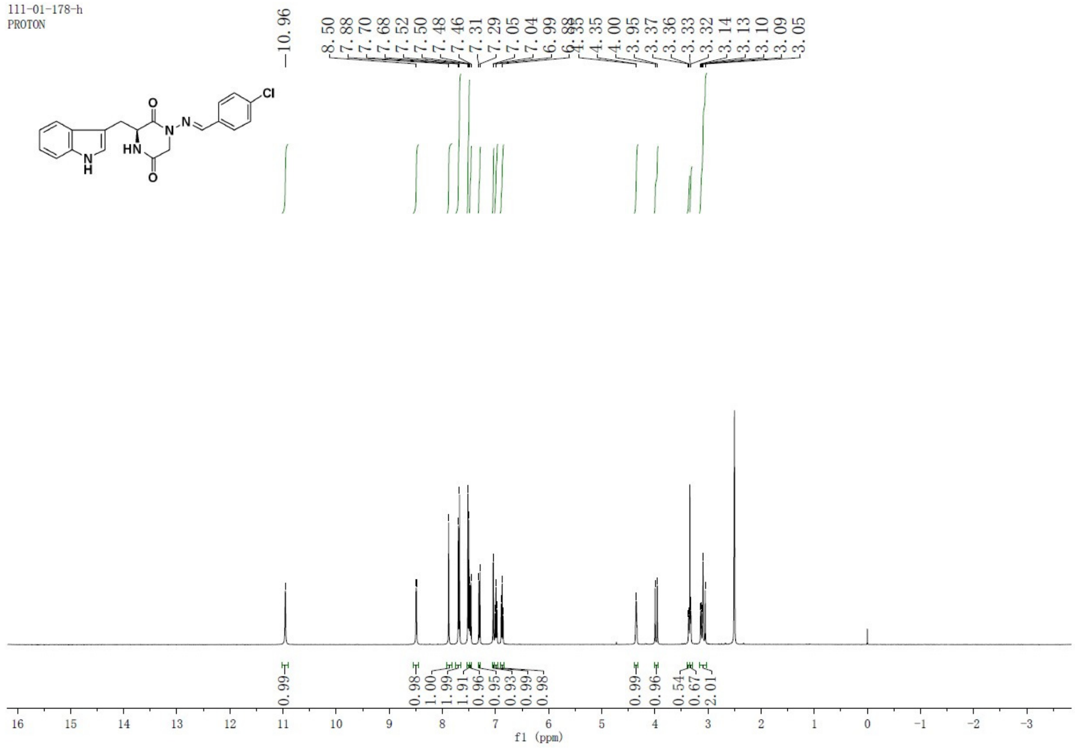


**Figure S15** $^{1}$H NMR (400 MHz, DMSO-*d₆*) of **8**

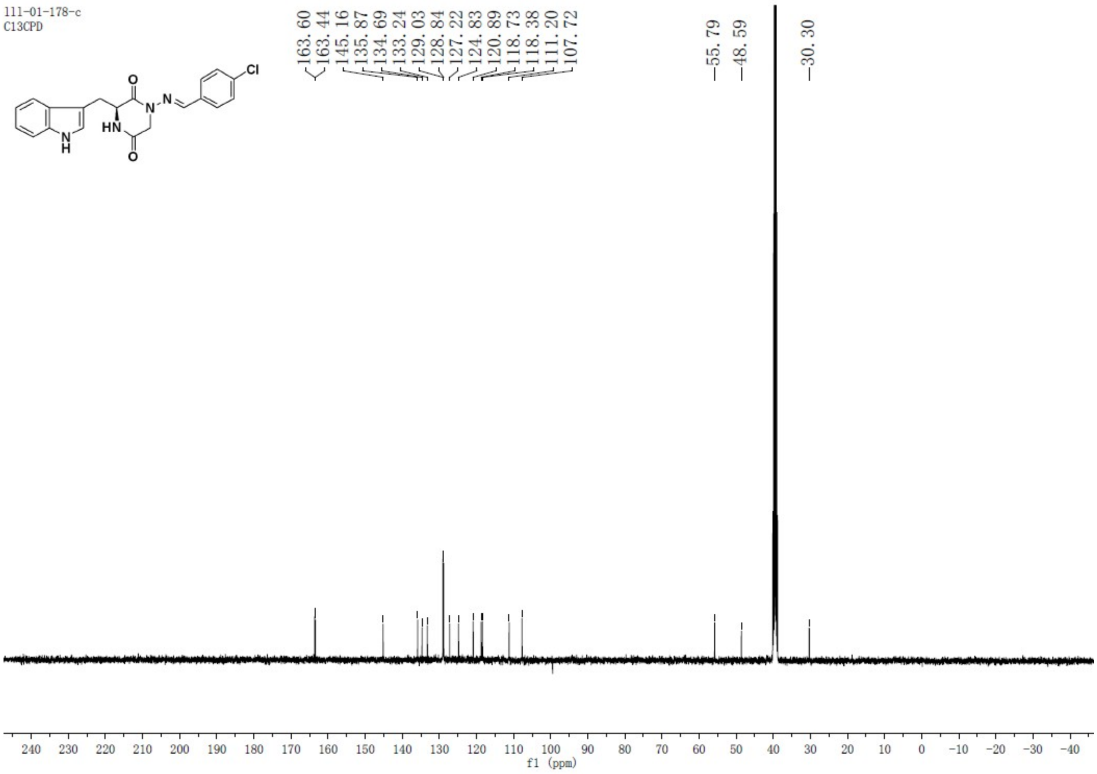


**Figure S16** $^{13}$C NMR (100 MHz, DMSO-*d₆*) of **8**

# 9

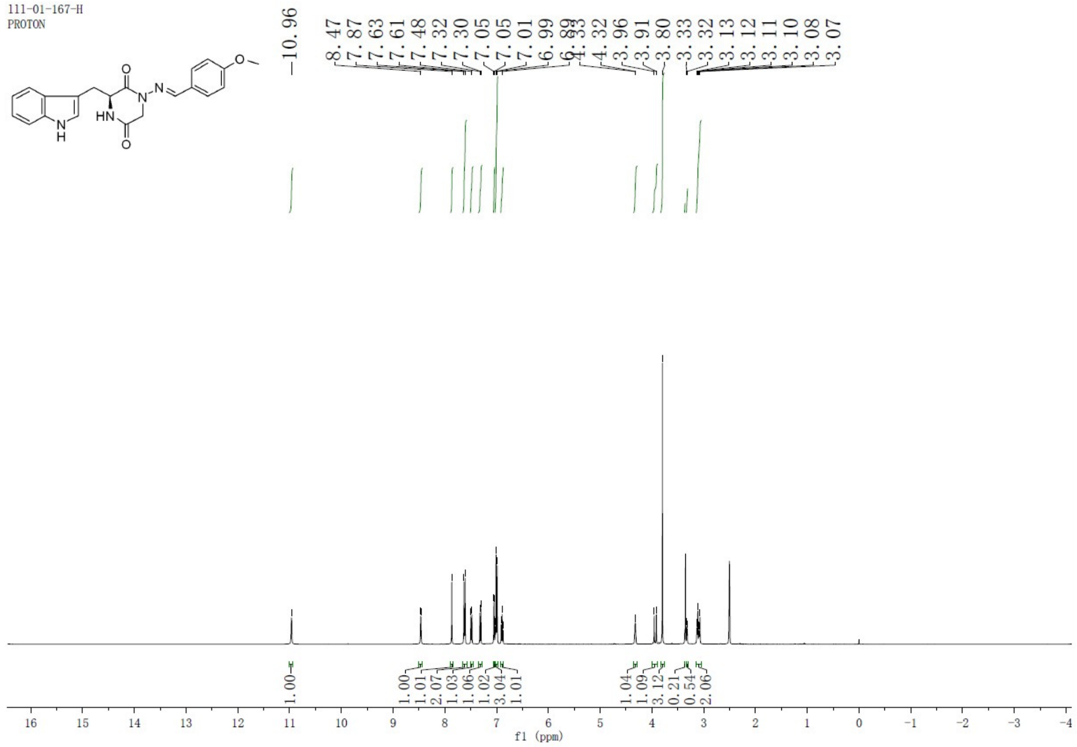


**Figure S17** $^{1}$H NMR (400 MHz, DMSO-$d_6$) of **9**

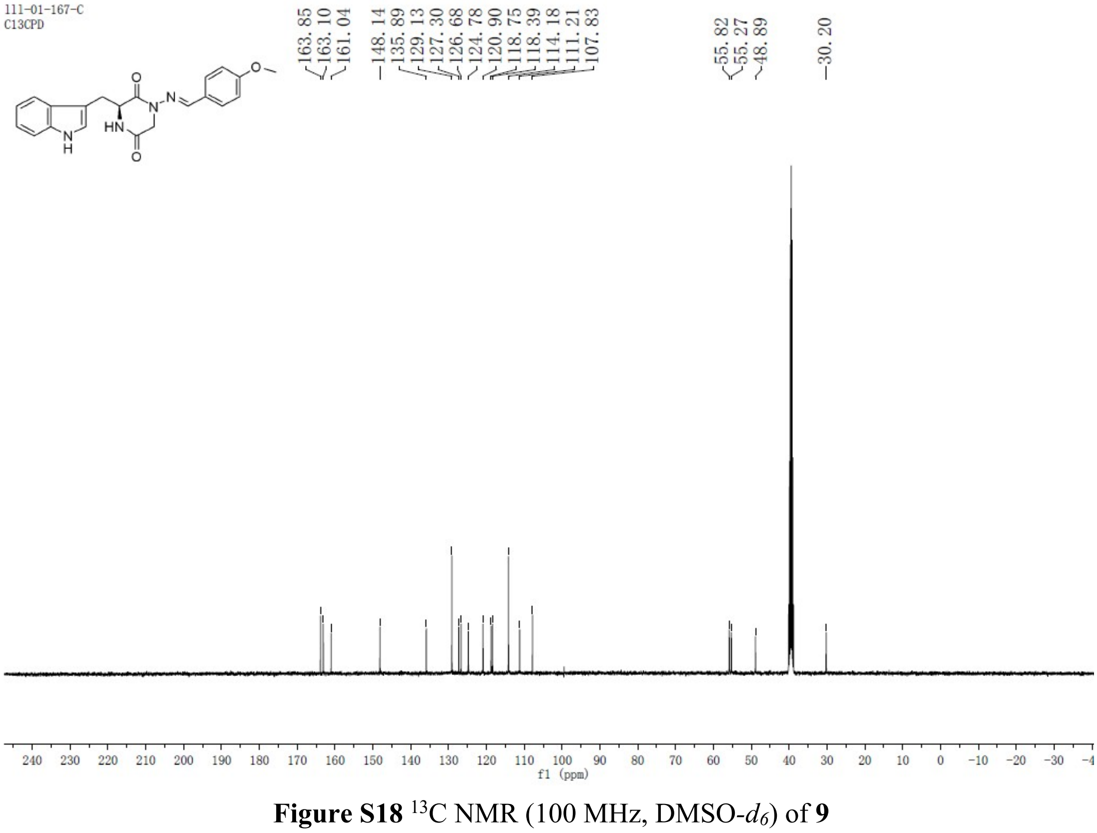


**Figure S18** $^{13}$C NMR (100 MHz, DMSO-$d_6$) of **9**

**10**

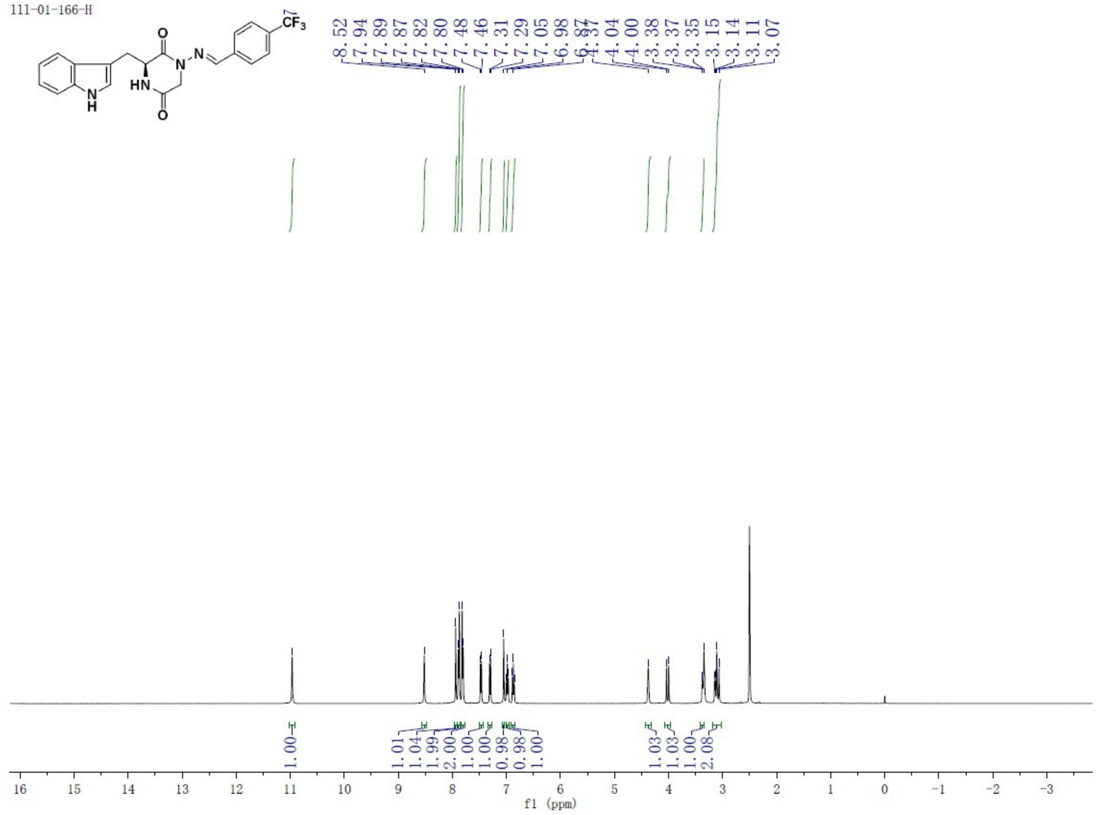


**Figure S19** $^{1}$H NMR (400 MHz, DMSO-*d*$_6$) of **10**

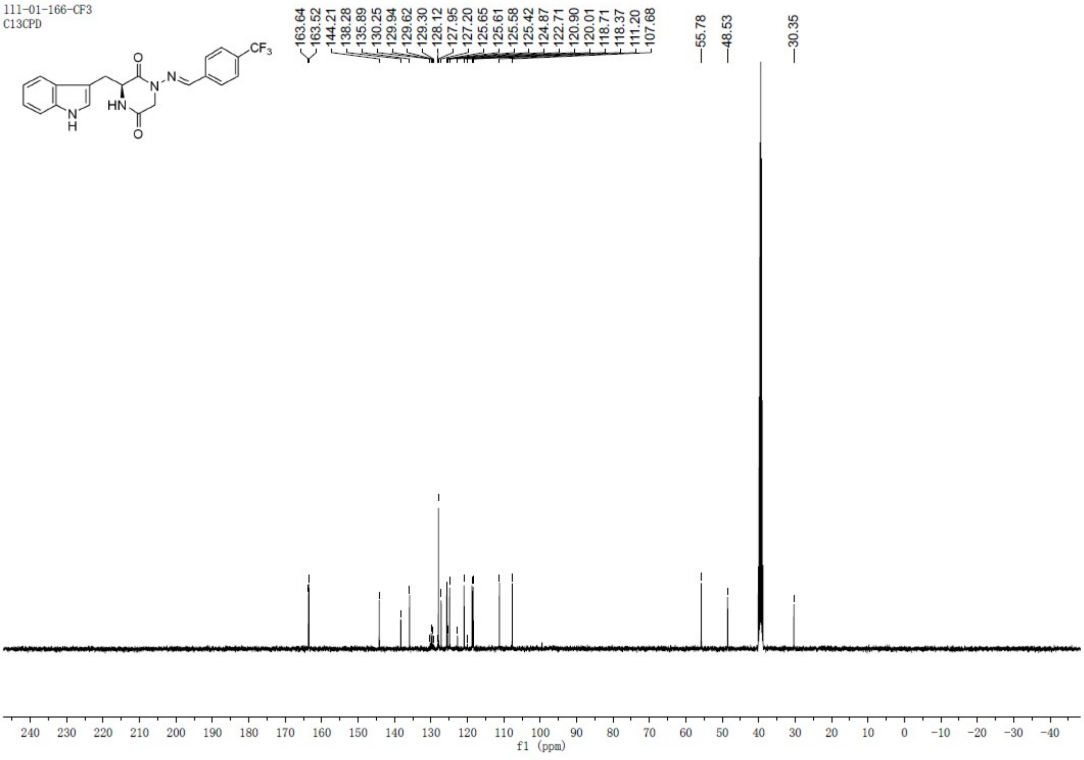


**Figure S20** $^{13}$C NMR (100 MHz, DMSO-*d*$_6$) of **10**

**11**

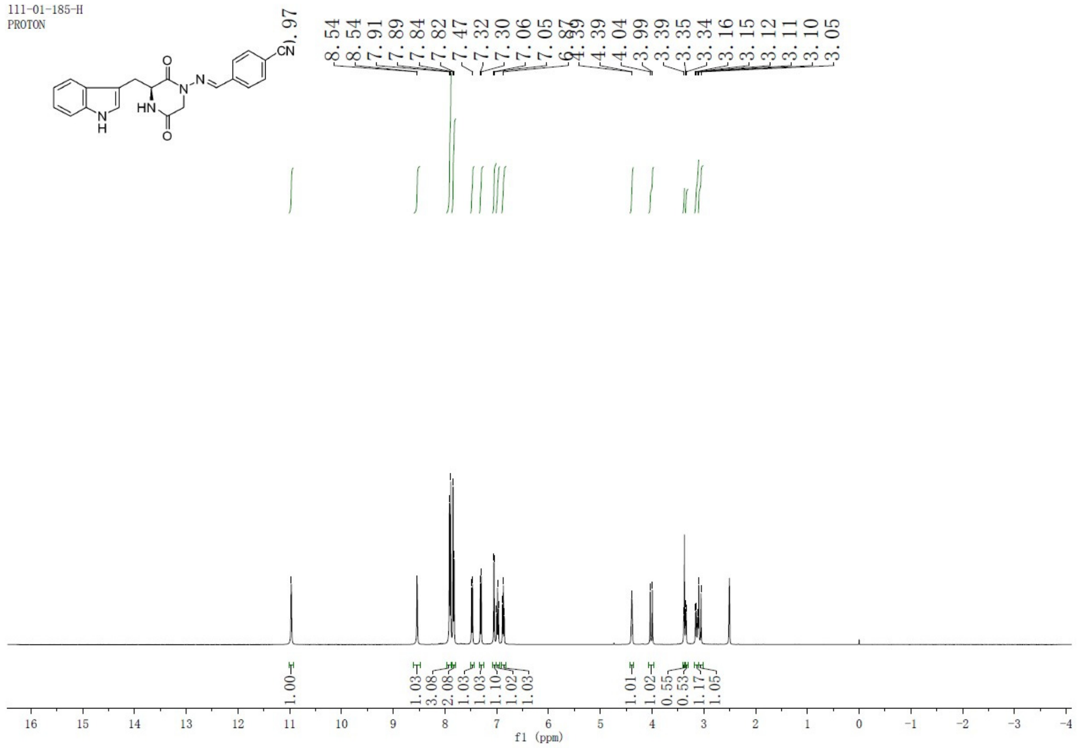


**Figure S21** $^{1}$H NMR (400 MHz, DMSO-$d_6$) of **11**

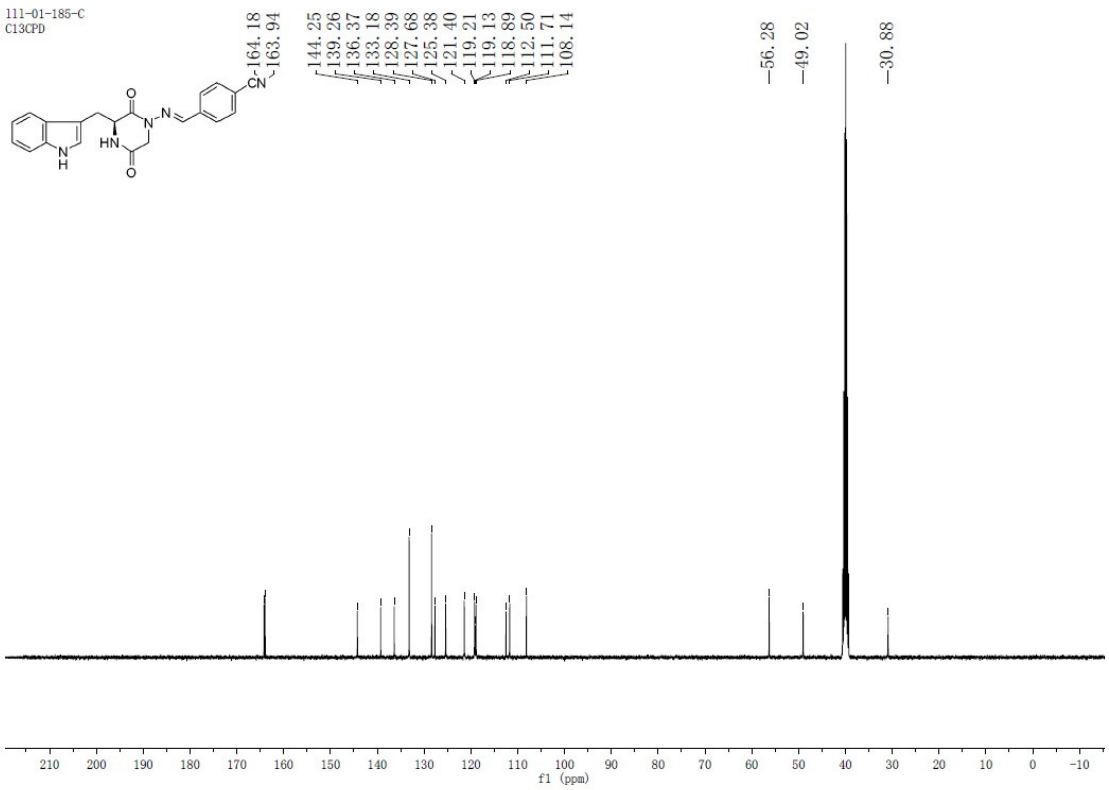


**Figure S22** $^{13}$C NMR (100 MHz, DMSO-$d_6$) of **11**

**12**

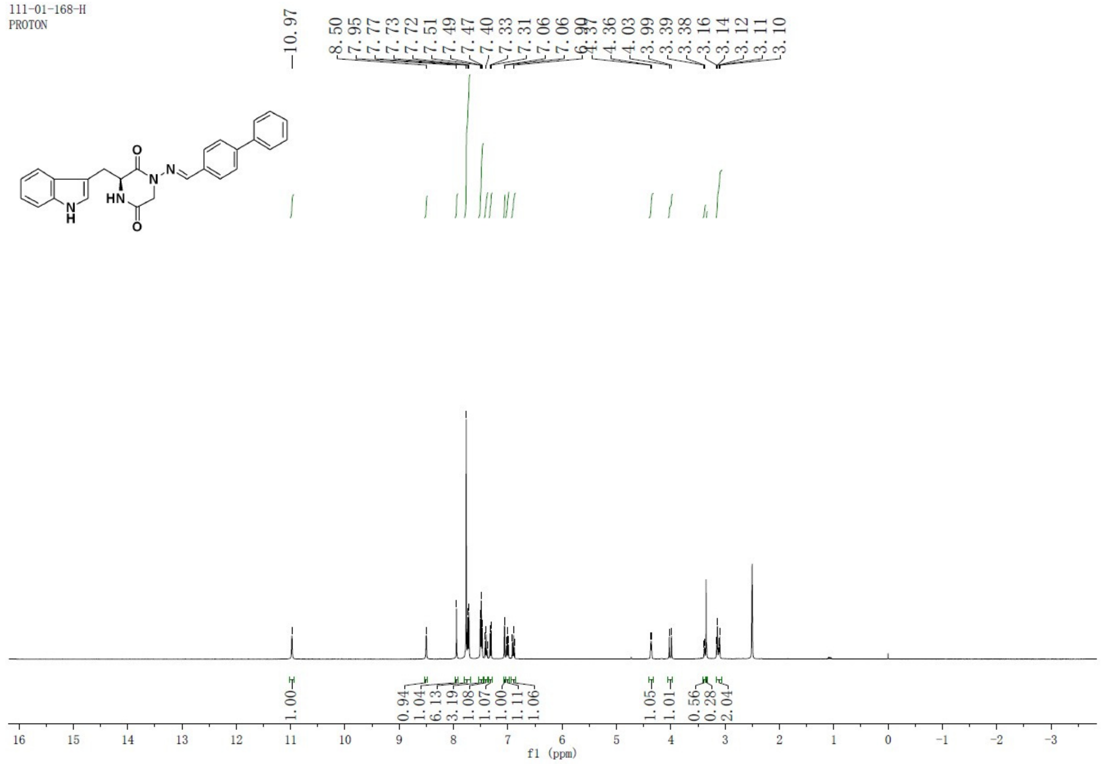


**Figure S23** $^{1}$H NMR (400 MHz, DMSO-$d_6$) of **12**

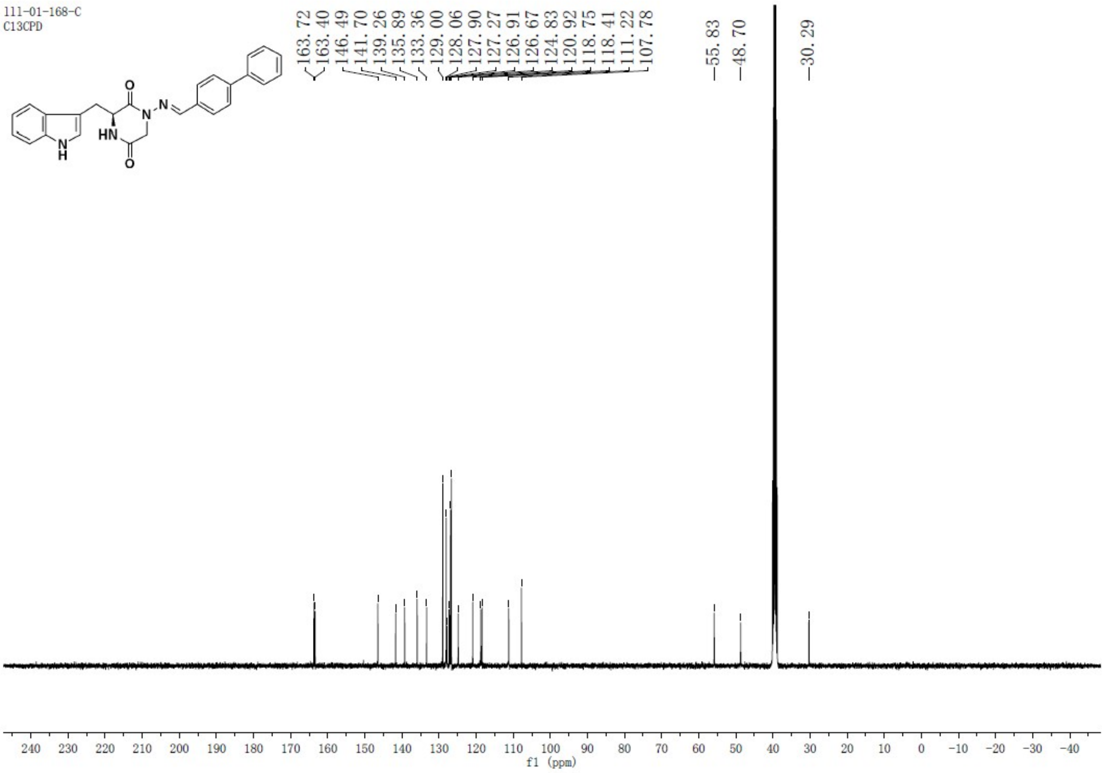


**Figure S24** $^{13}$C NMR (100 MHz, DMSO-$d_6$) of **12**

**13**

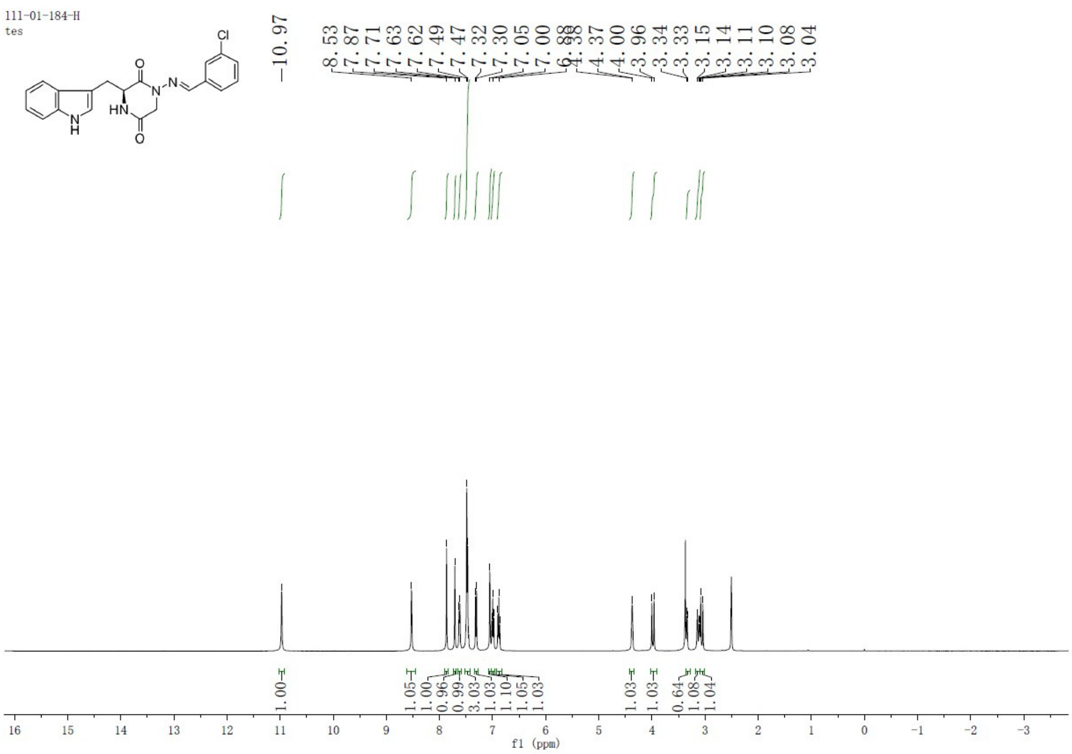

**Figure S25** $^{1}$H NMR (400 MHz, DMSO-$d_6$) of **13**

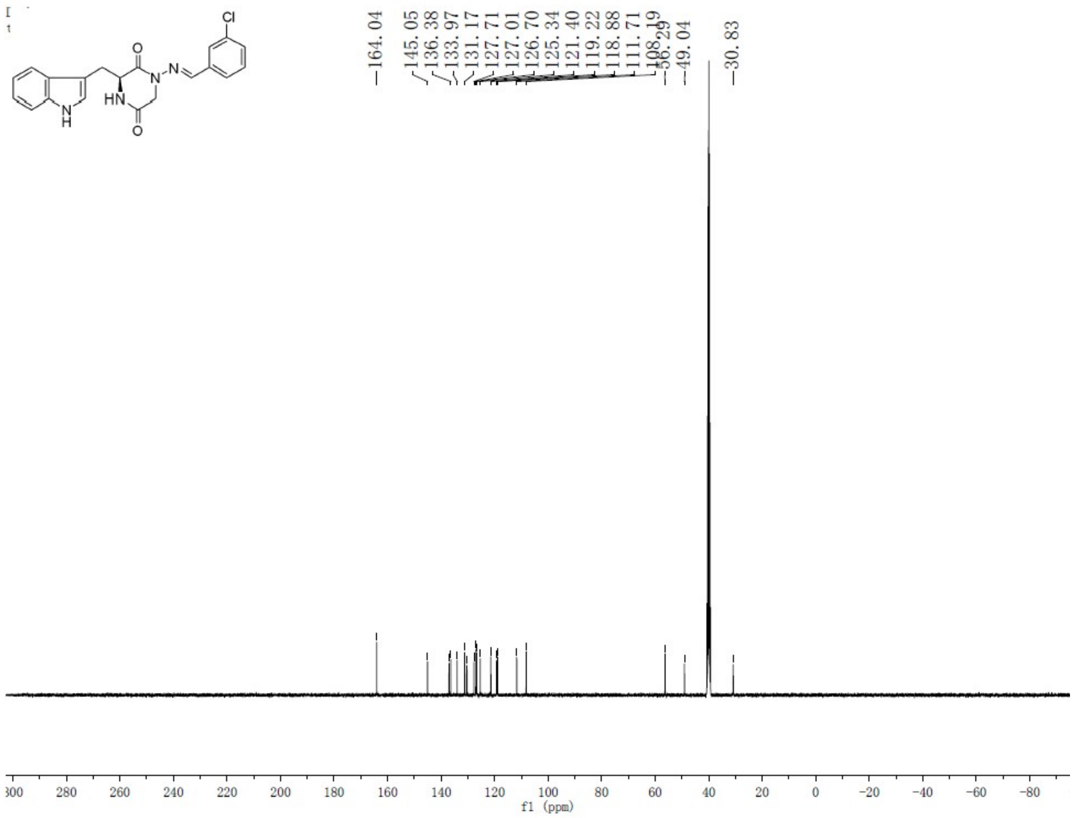

**Figure S26** $^{13}$C NMR (100 MHz, DMSO-$d_6$) of **13**

# 14

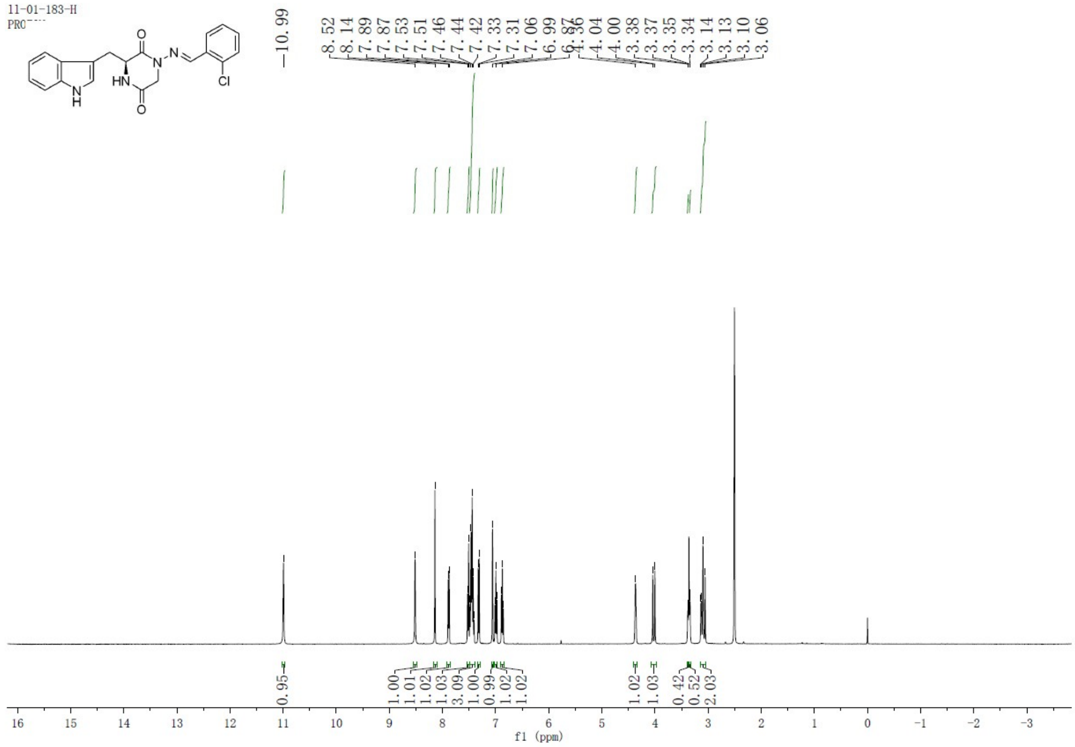


**Figure S27** $^{1}$H NMR (400 MHz, DMSO-$d_6$) of **14**

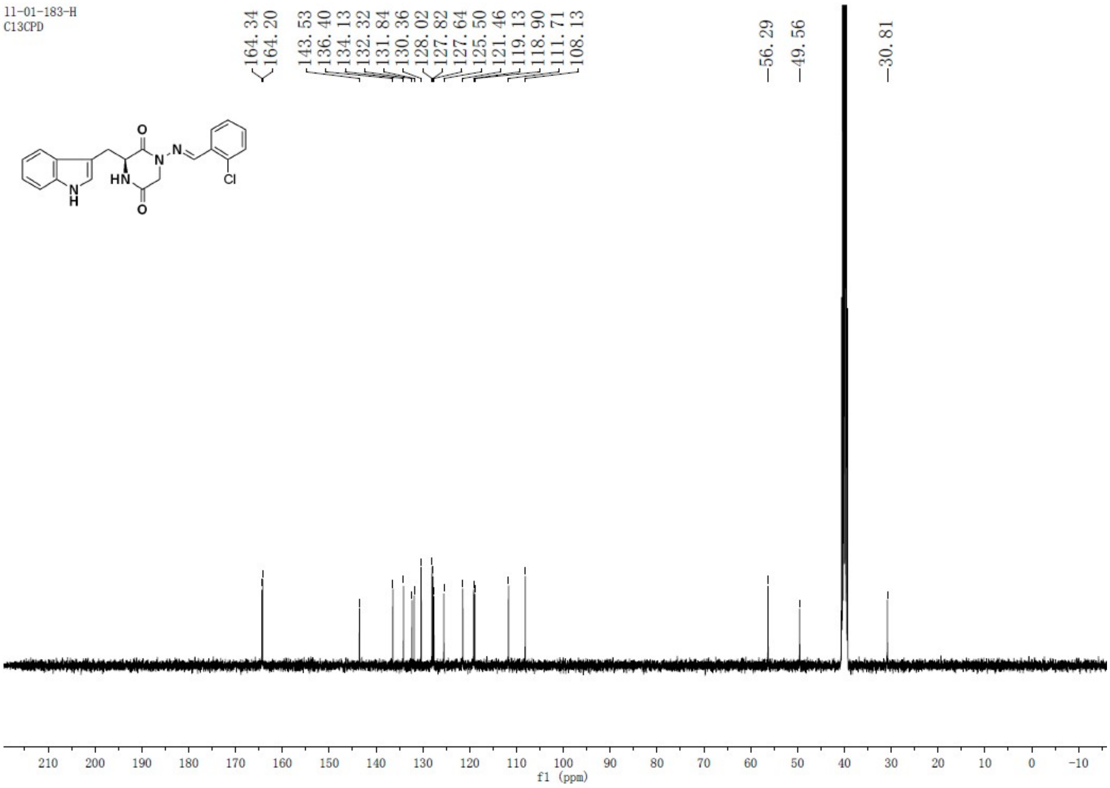


**Figure S28** $^{13}$C NMR (100 MHz, DMSO-$d_6$) of **14**

**15**

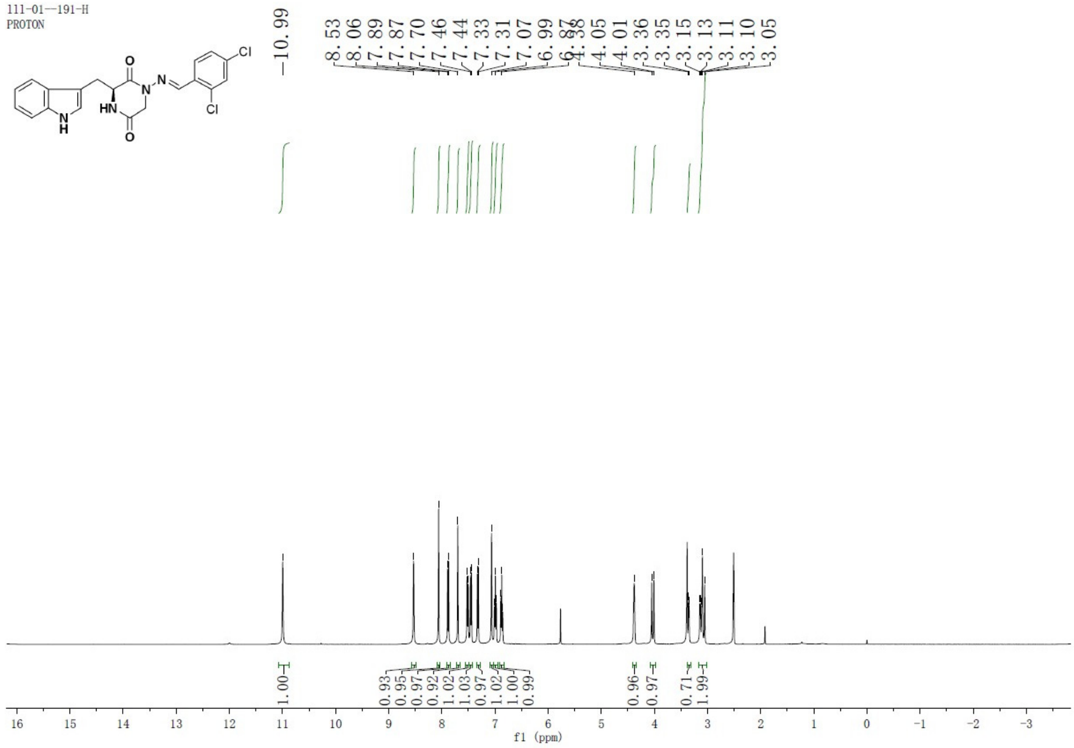

Figure S29 $^{1}$H NMR (400 MHz, DMSO-$d_6$) of **15**

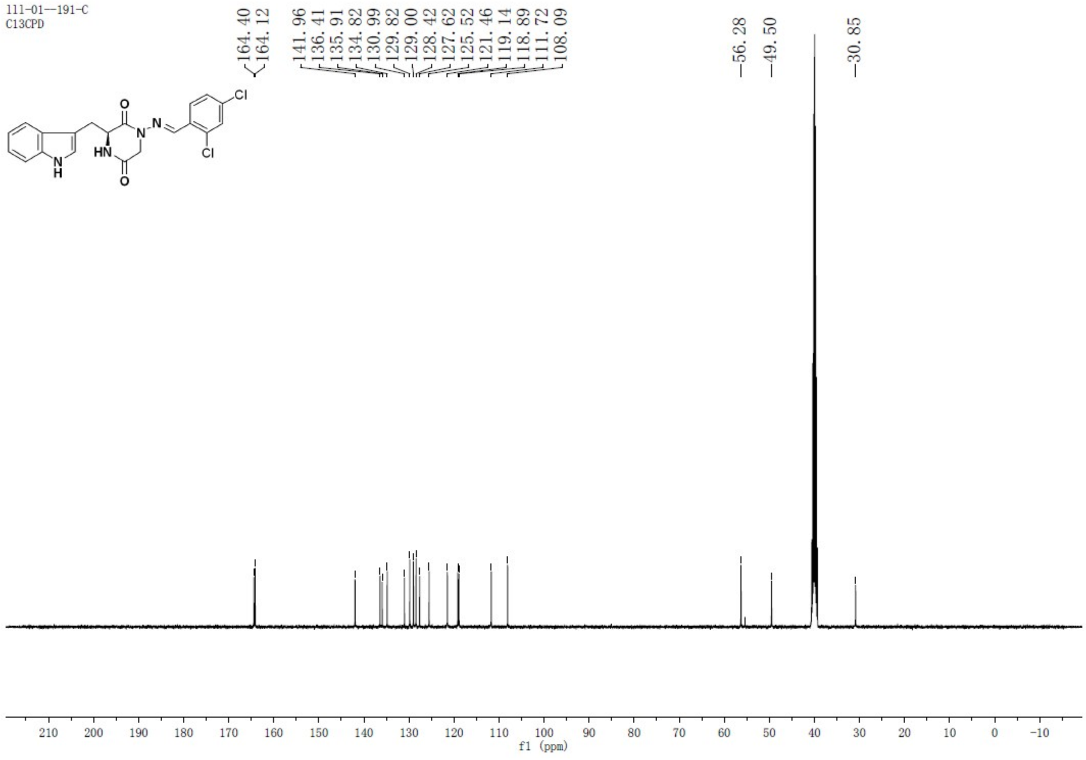

Figure S30 $^{13}$C NMR (100 MHz, DMSO-$d_6$) of **15**

**16**

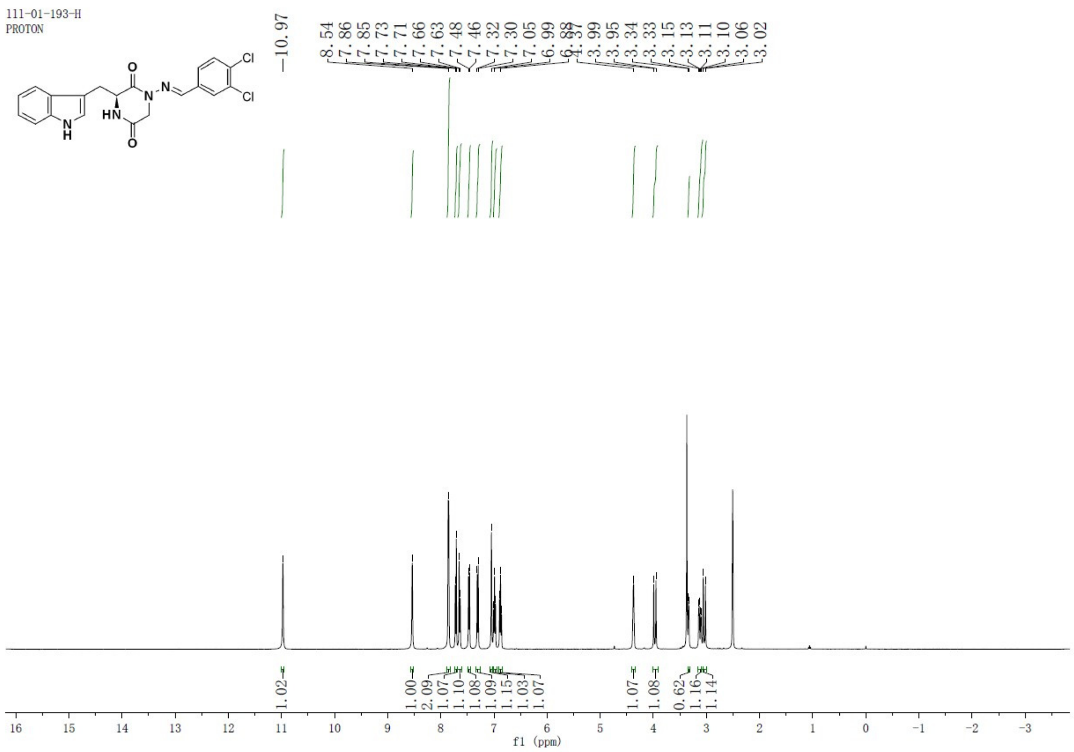


**Figure S31** $^1$H NMR (400 MHz, DMSO-$d_6$) of **16**

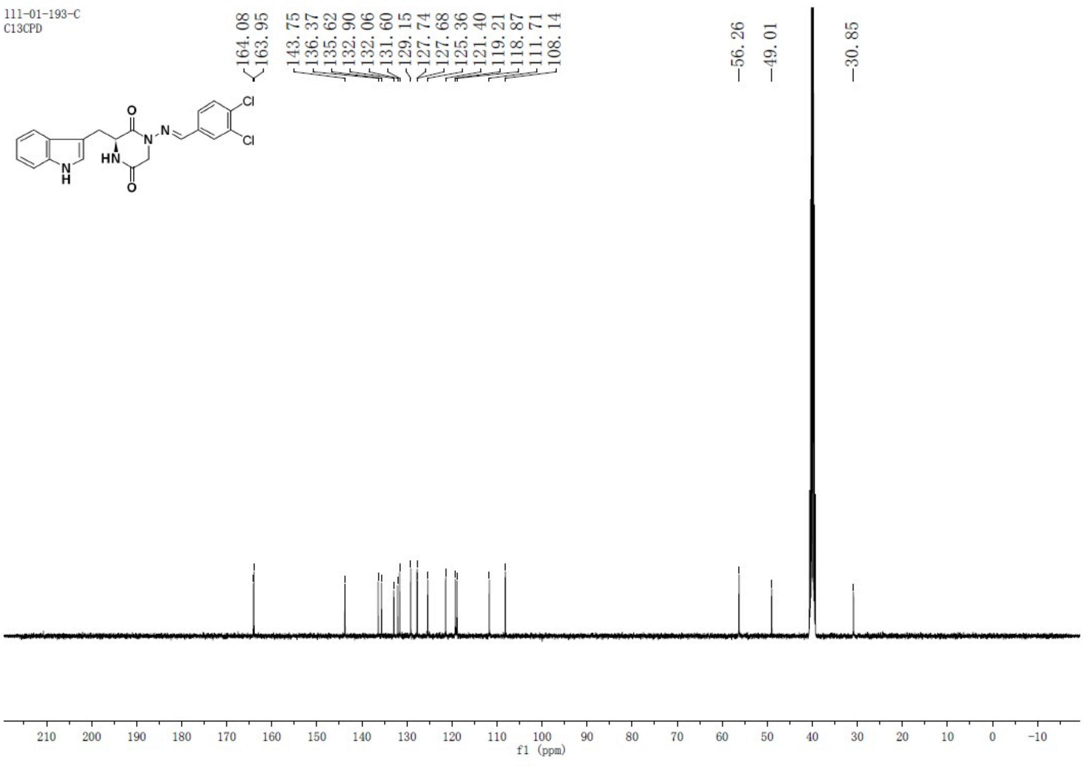


**Figure S32** $^{13}$C NMR (100 MHz, DMSO-$d_6$) of **16**

**17**

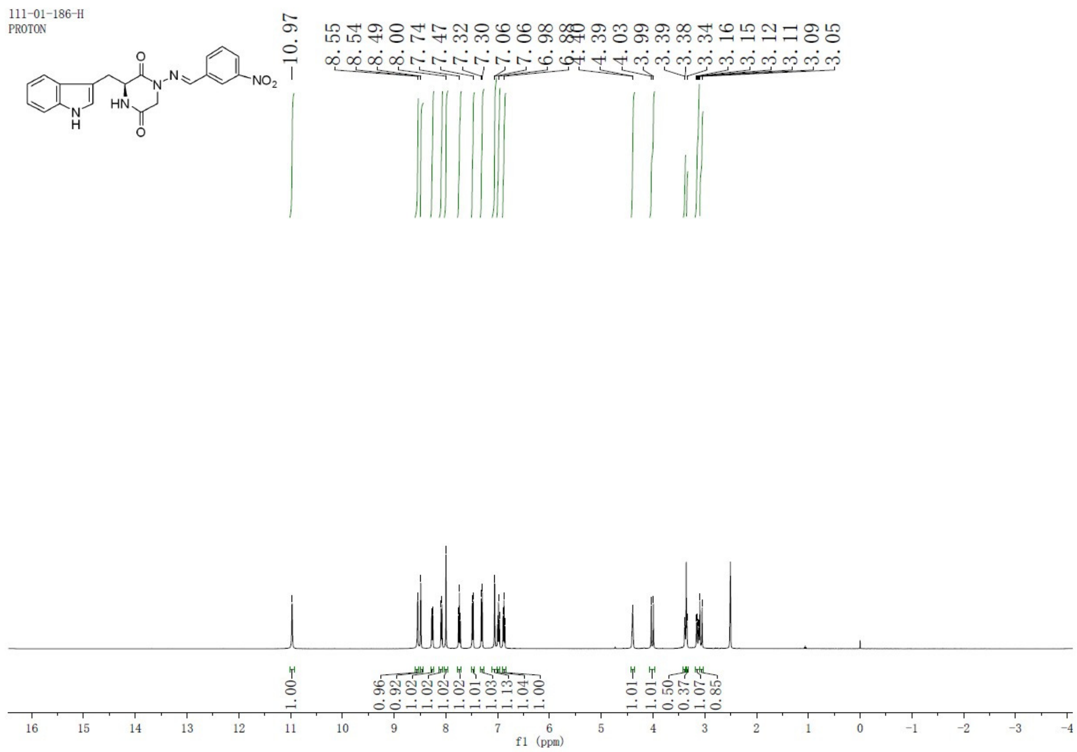


**Figure S33** $^{1}$H NMR (400 MHz, DMSO-$d_6$) of **17**

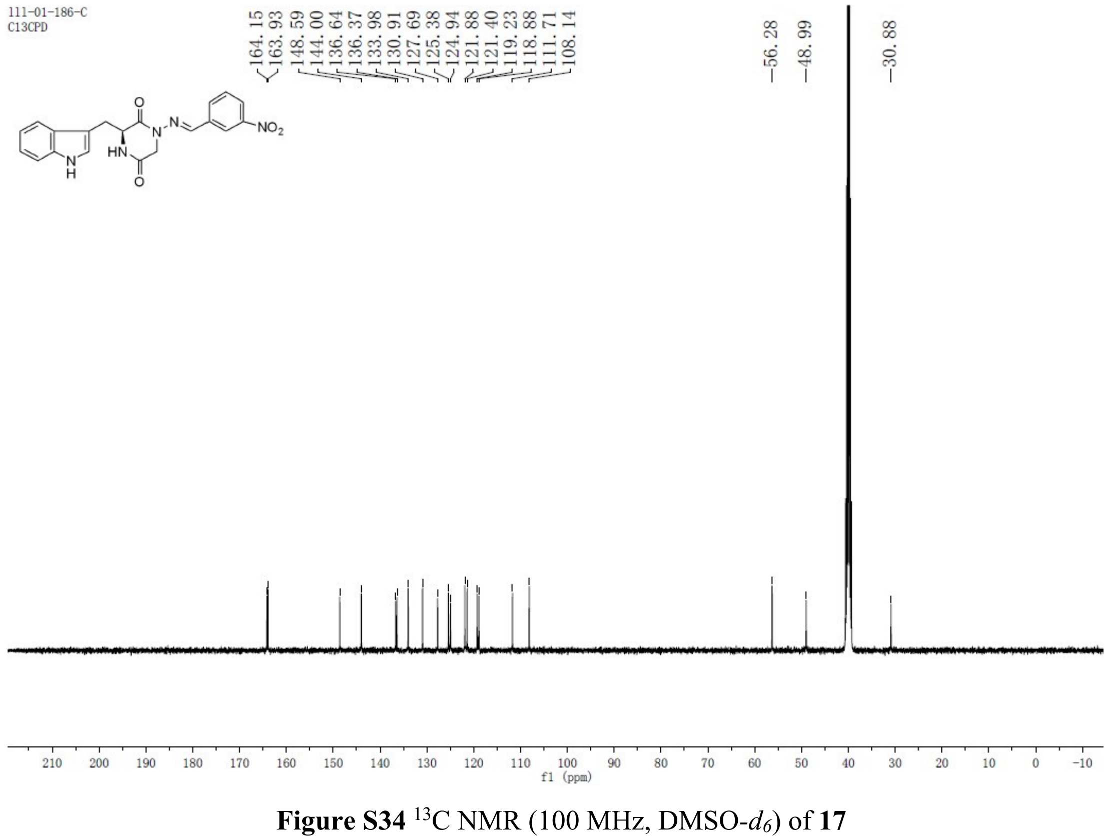


**Figure S34** $^{13}$C NMR (100 MHz, DMSO-$d_6$) of **17**

**18**

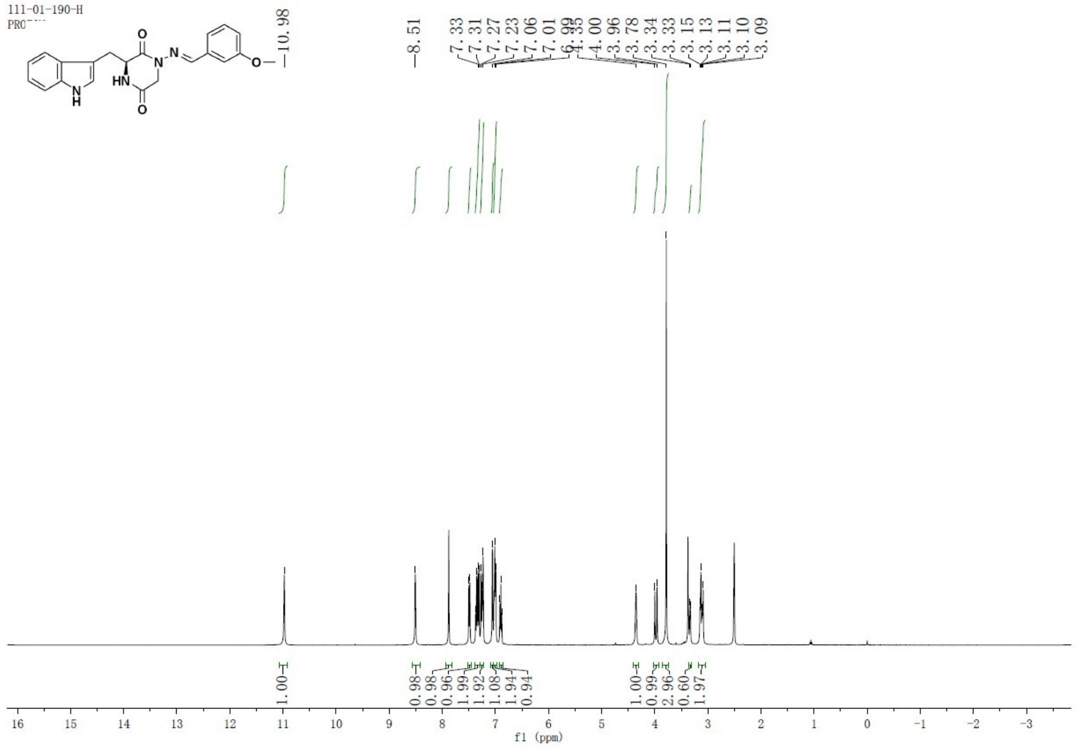


**Figure S35** $^{1}$H NMR (400 MHz, DMSO-$d_6$) of **18**

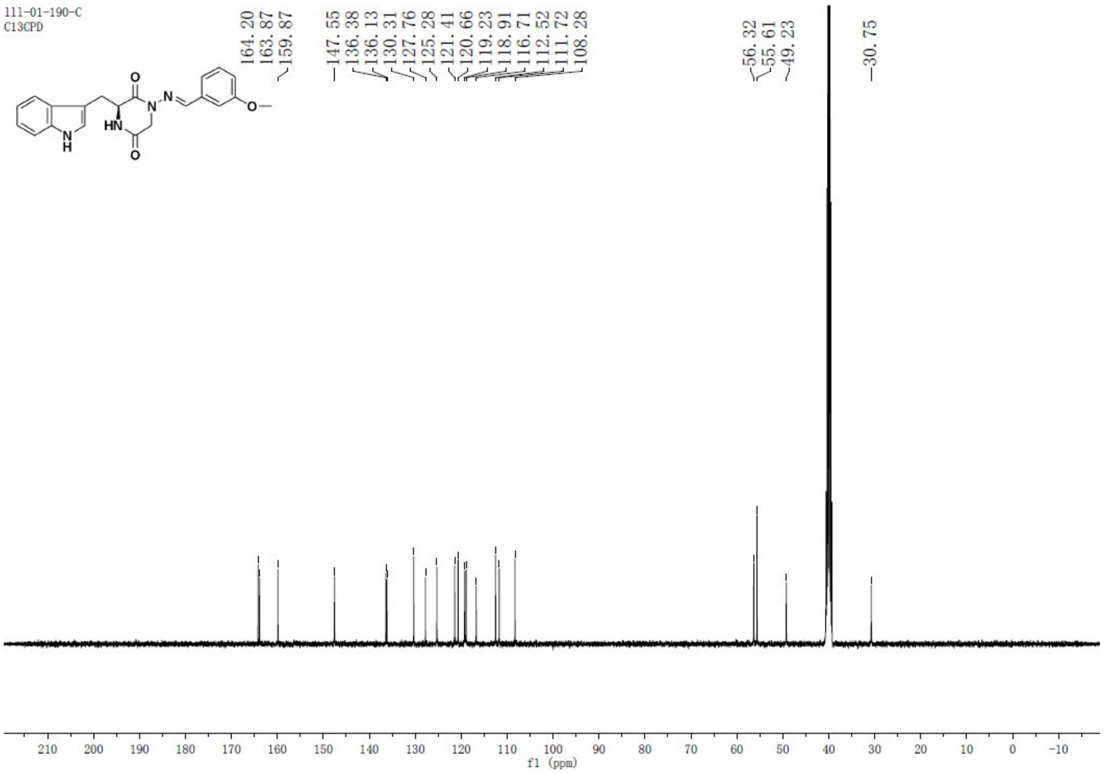


**Figure S36** $^{13}$C NMR (100 MHz, DMSO-$d_6$) of **18**

**19**

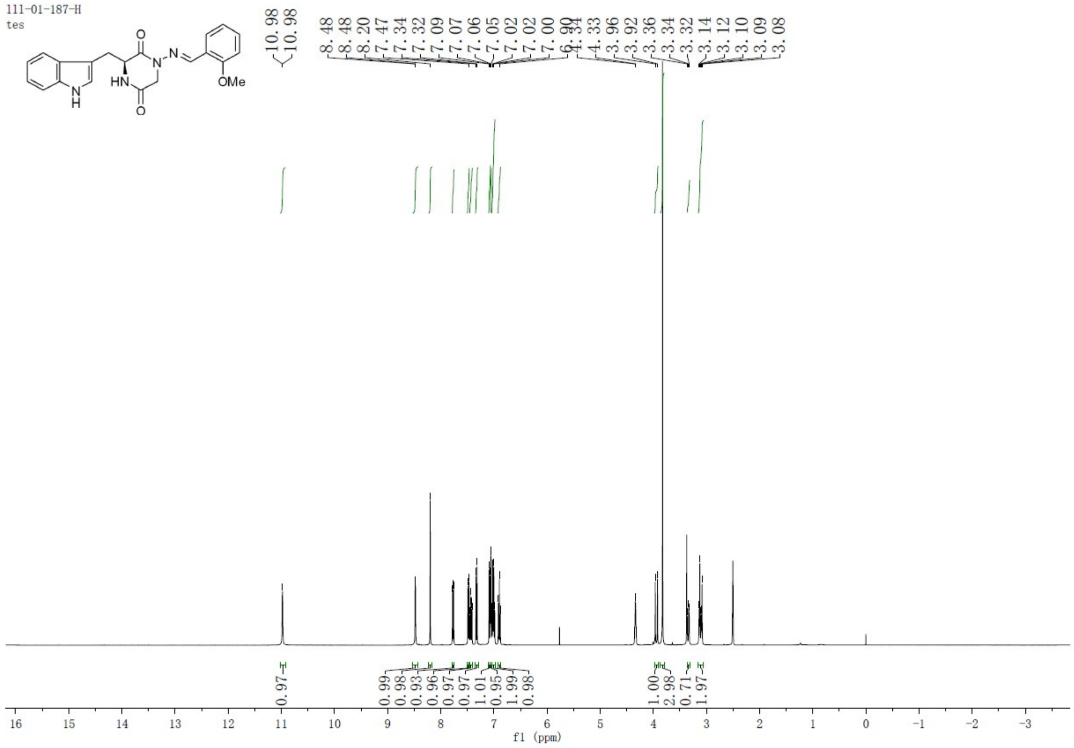


**Figure S37** $^{1}$H NMR (400 MHz, DMSO-$d_6$) of **19**

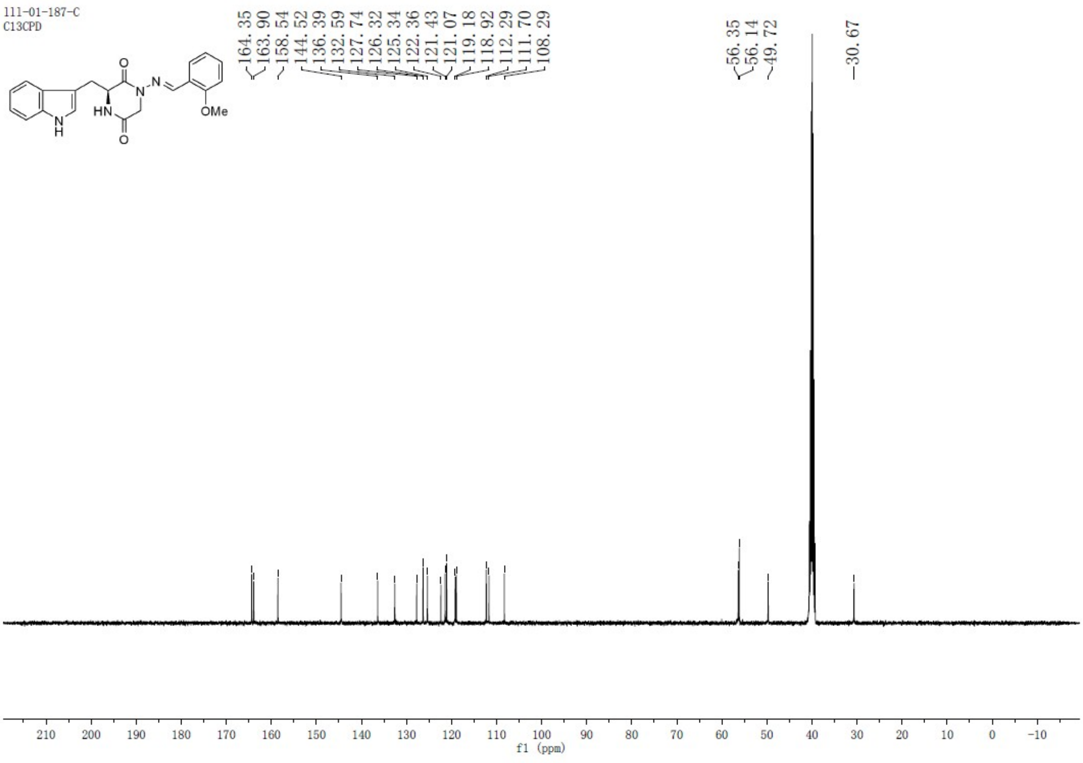


**Figure S38** $^{13}$C NMR (100 MHz, DMSO-$d_6$) of **19**

# 20

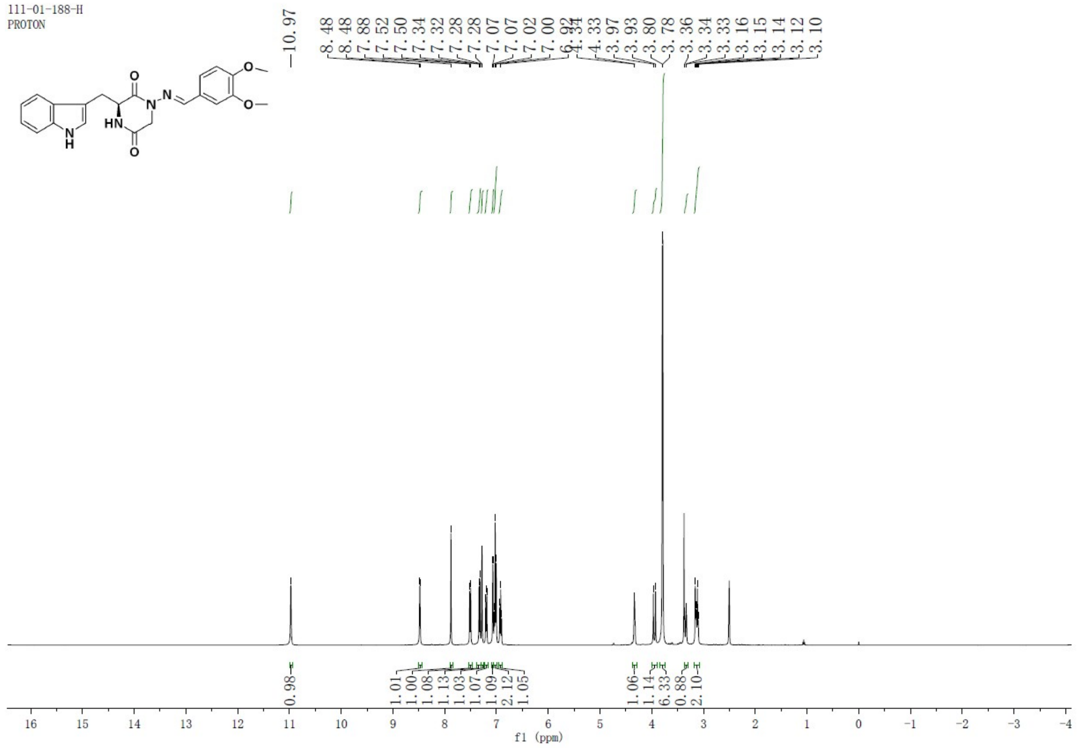


**Figure S39** $^{1}$H NMR (400 MHz, DMSO-$d_6$) of **20**

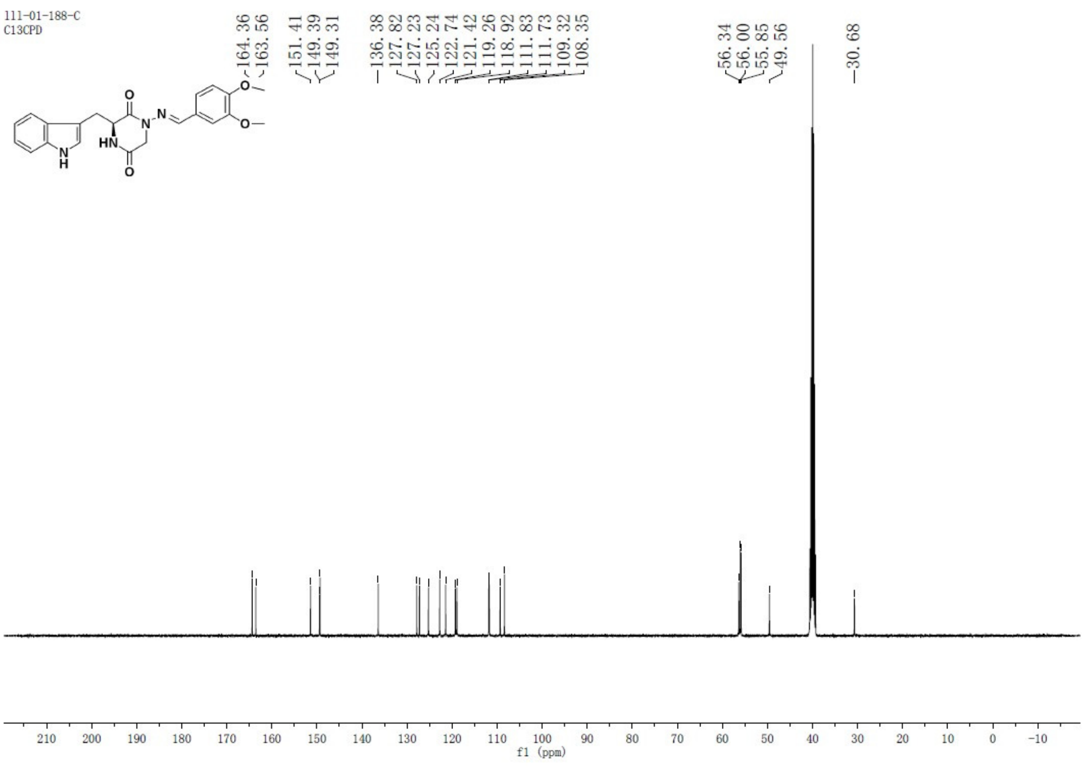


**Figure S40** $^{13}$C NMR (100 MHz, DMSO-$d_6$) of **20**

# 21

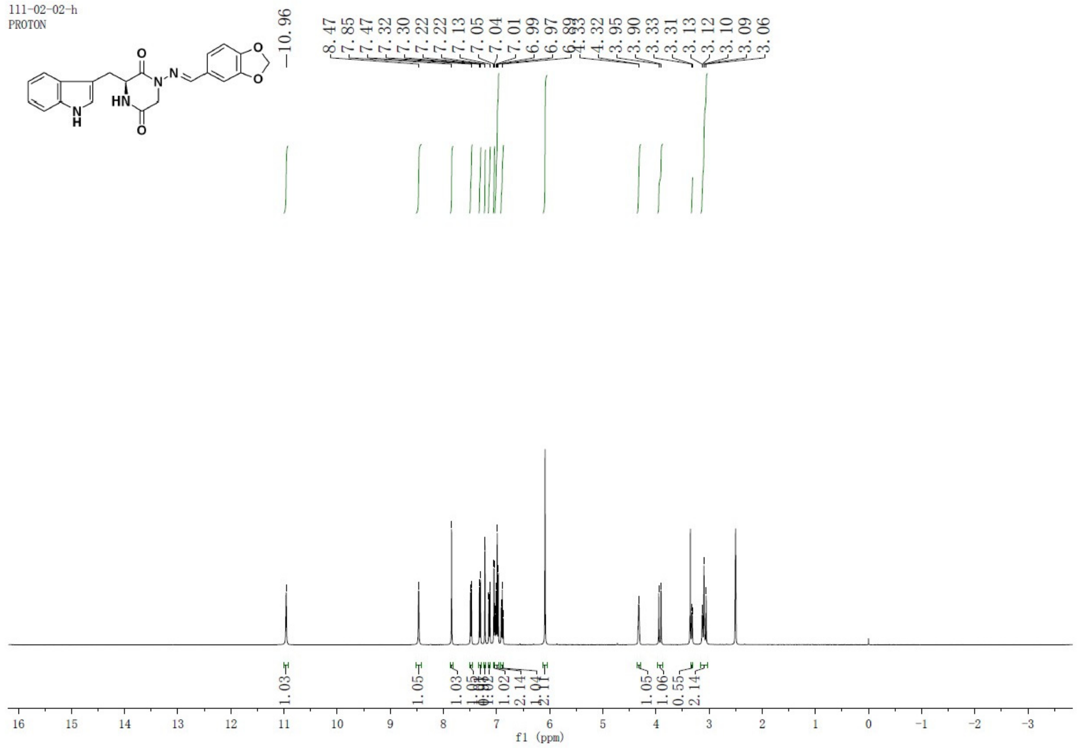


**Figure S41** $^{1}$H NMR (400 MHz, DMSO-$d_6$) of **21**

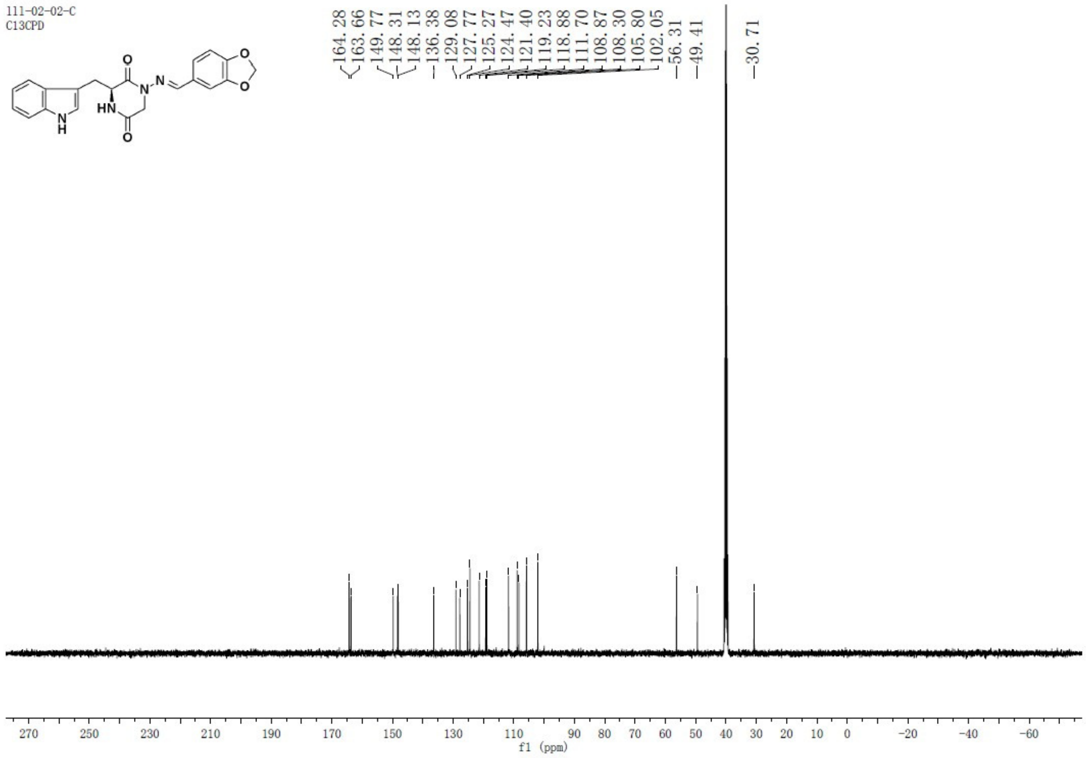


**Figure S42** $^{13}$C NMR (100 MHz, DMSO-$d_6$) of **21**

# 22

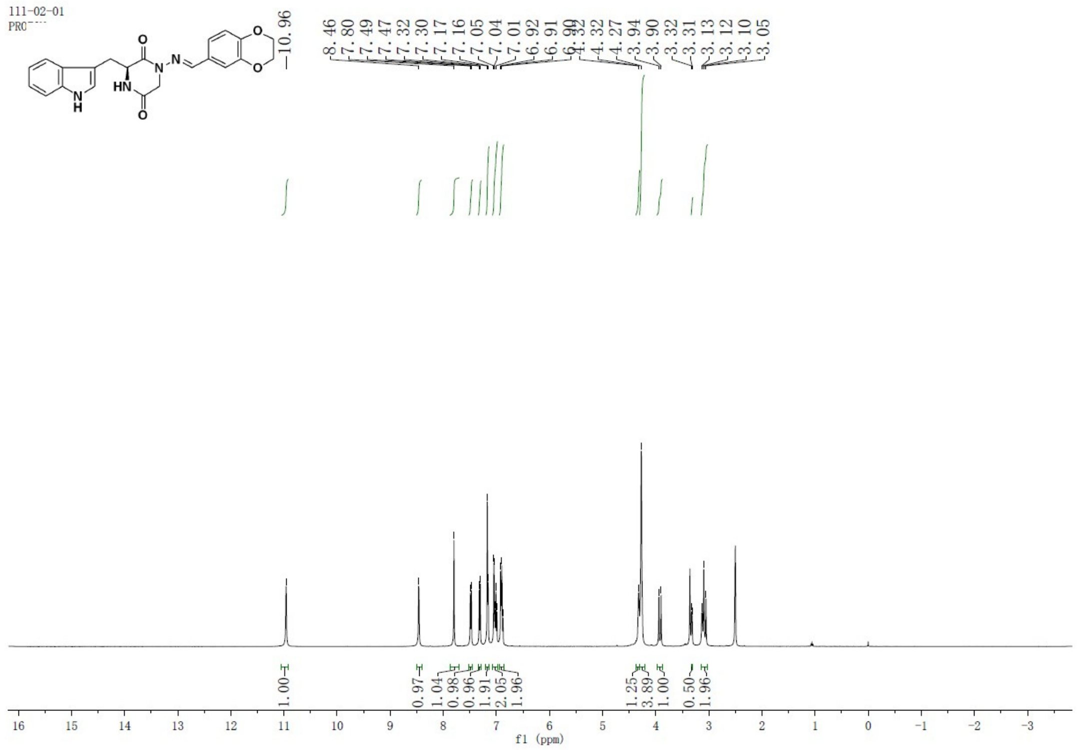


**Figure S43** $^{1}$H NMR (400 MHz, DMSO-$d_6$) of **22**

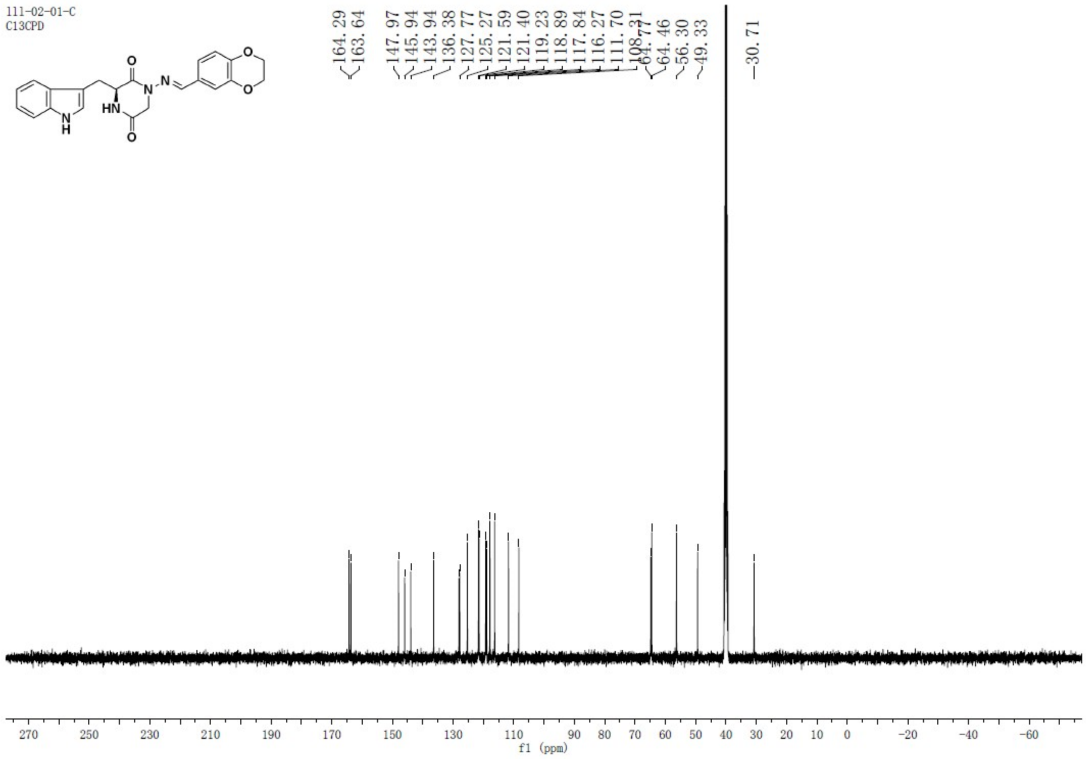


**Figure S44** $^{13}$C NMR (100 MHz, DMSO-$d_6$) of **22**

# 23

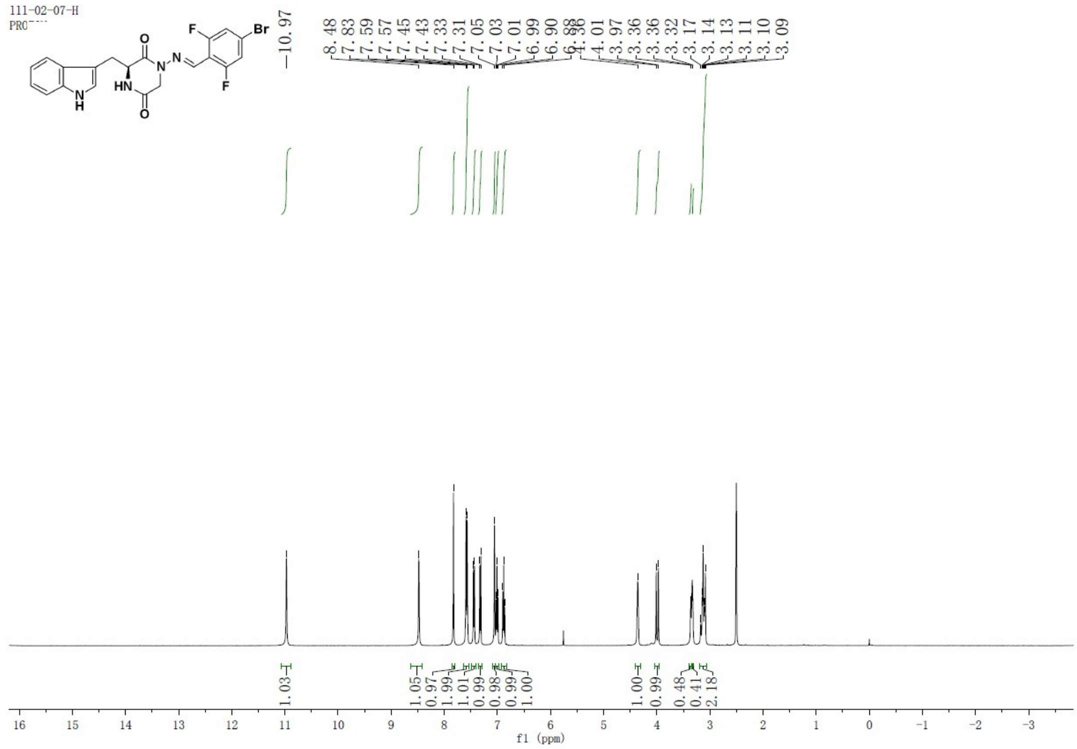

**Figure S45** $^{1}$H NMR (400 MHz, DMSO-$d_6$) of **23**

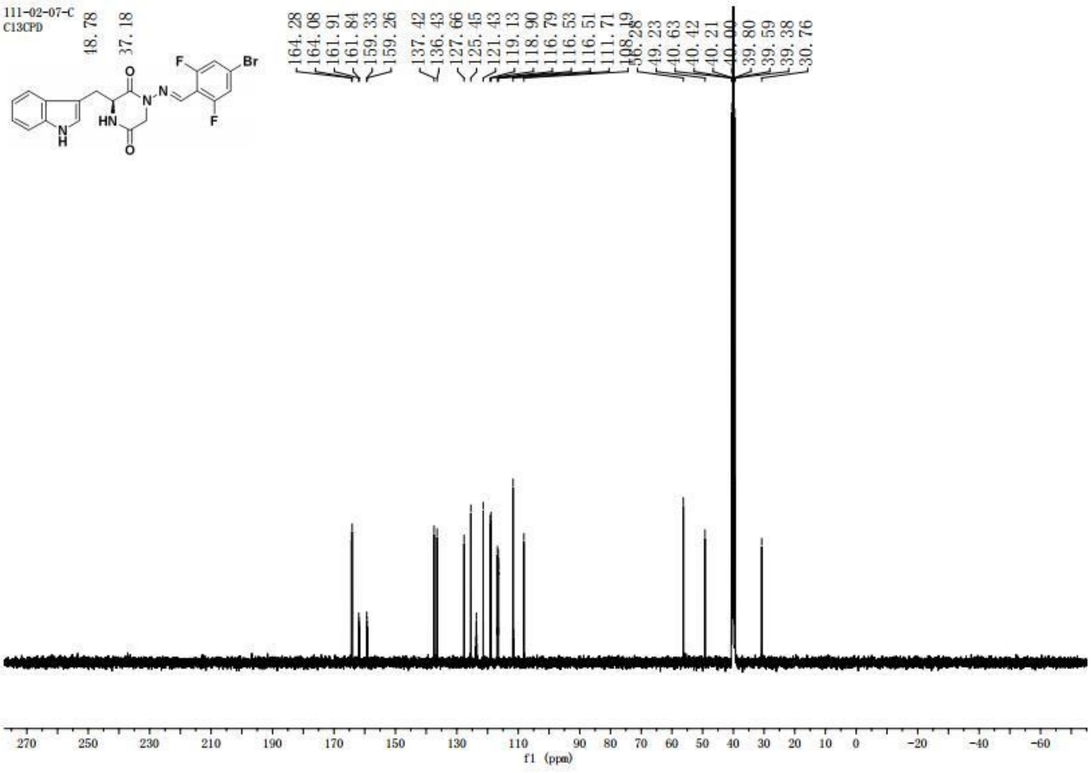

**Figure S46** $^{13}$C NMR (100 MHz, DMSO-$d_6$) of **23**

**24**

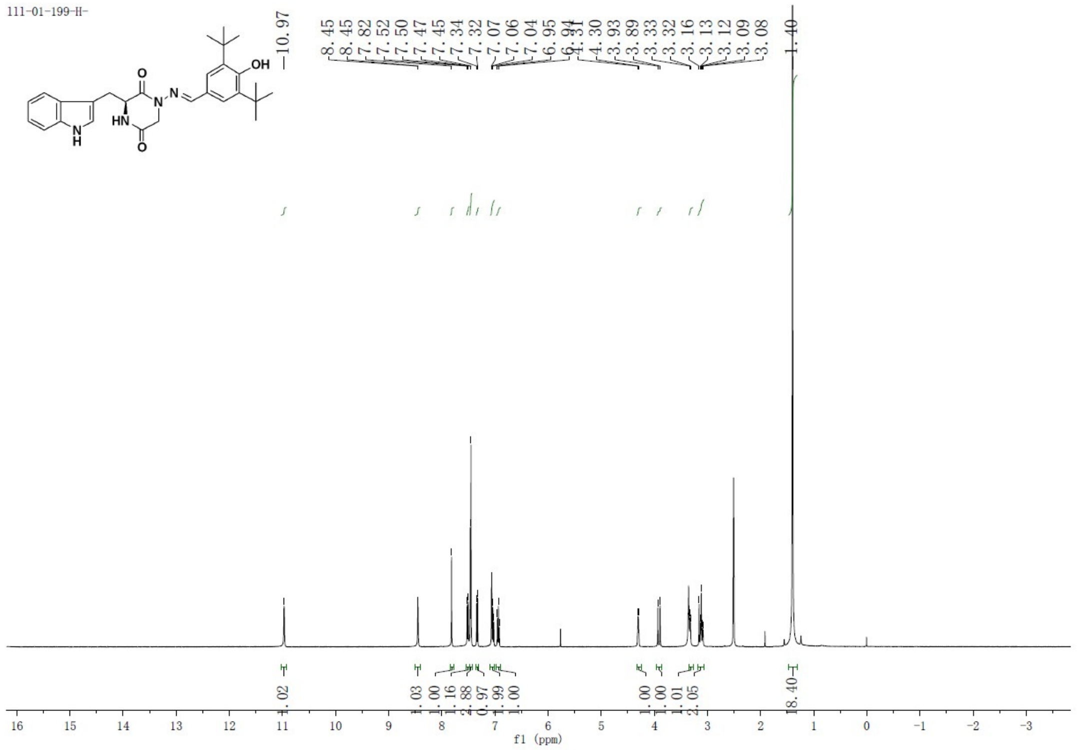

**Figure S47** $^{1}H$ NMR (400 MHz, DMSO-$d_6$) of **24**

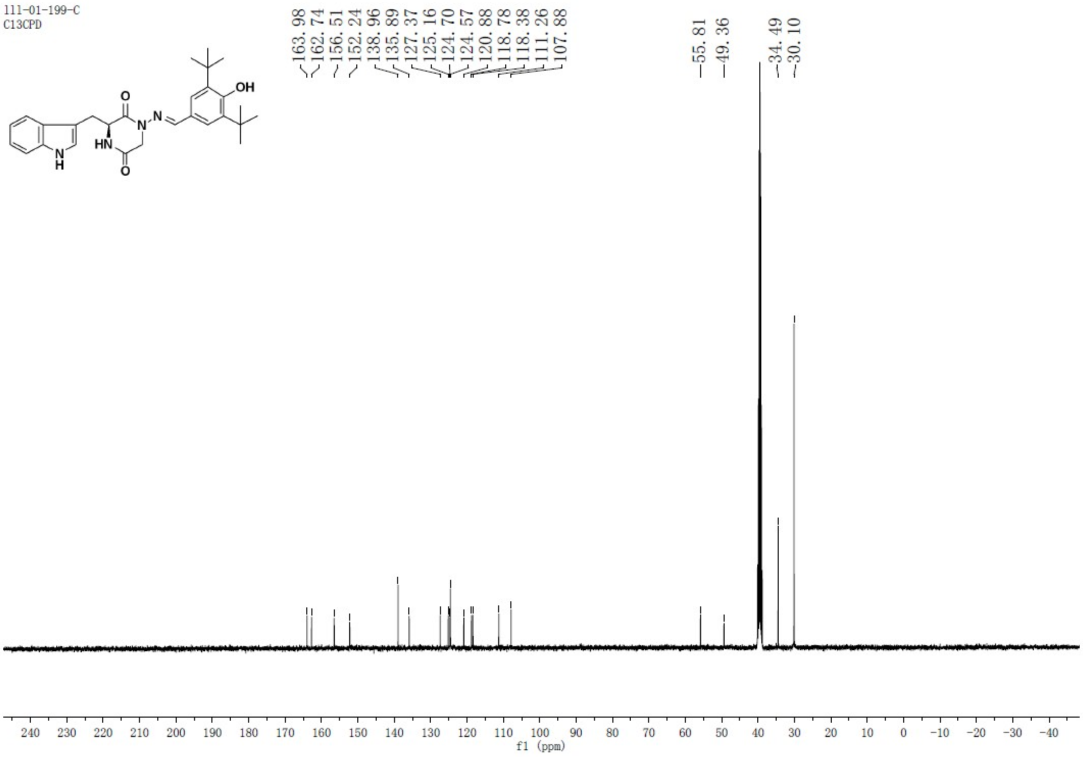

**Figure S48** $^{13}C$ NMR (100 MHz, DMSO-$d_6$) of **24**

**25**

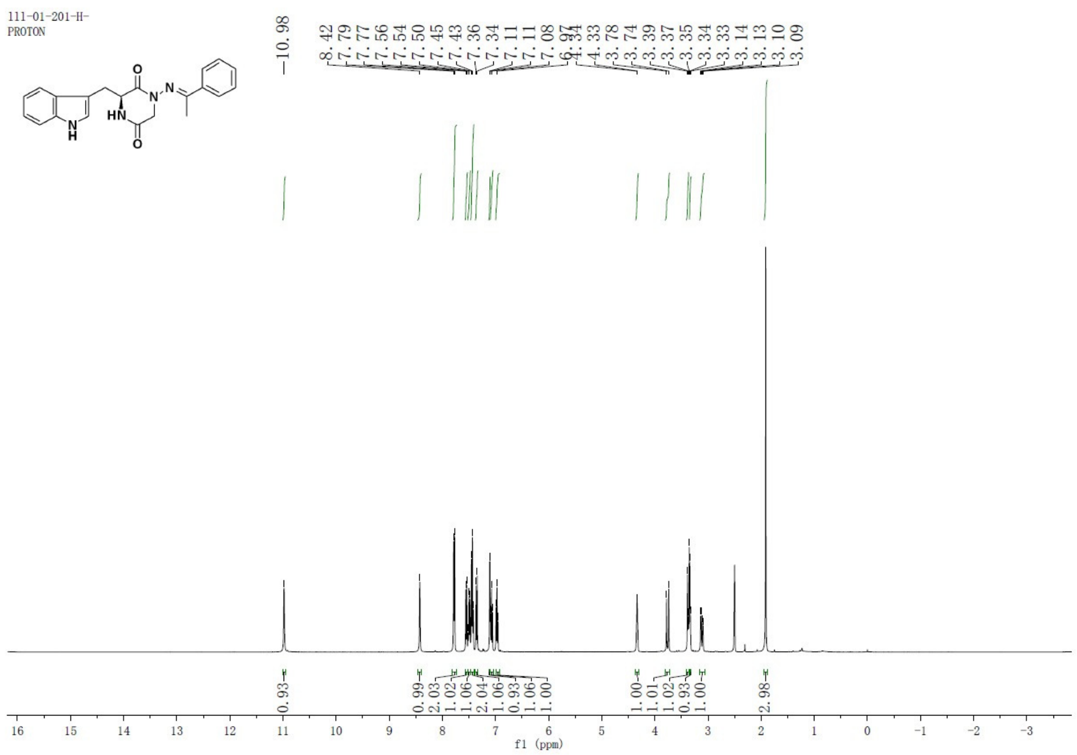


**Figure S49** $^{1}$H NMR (400 MHz, DMSO-$d_6$) of **25**

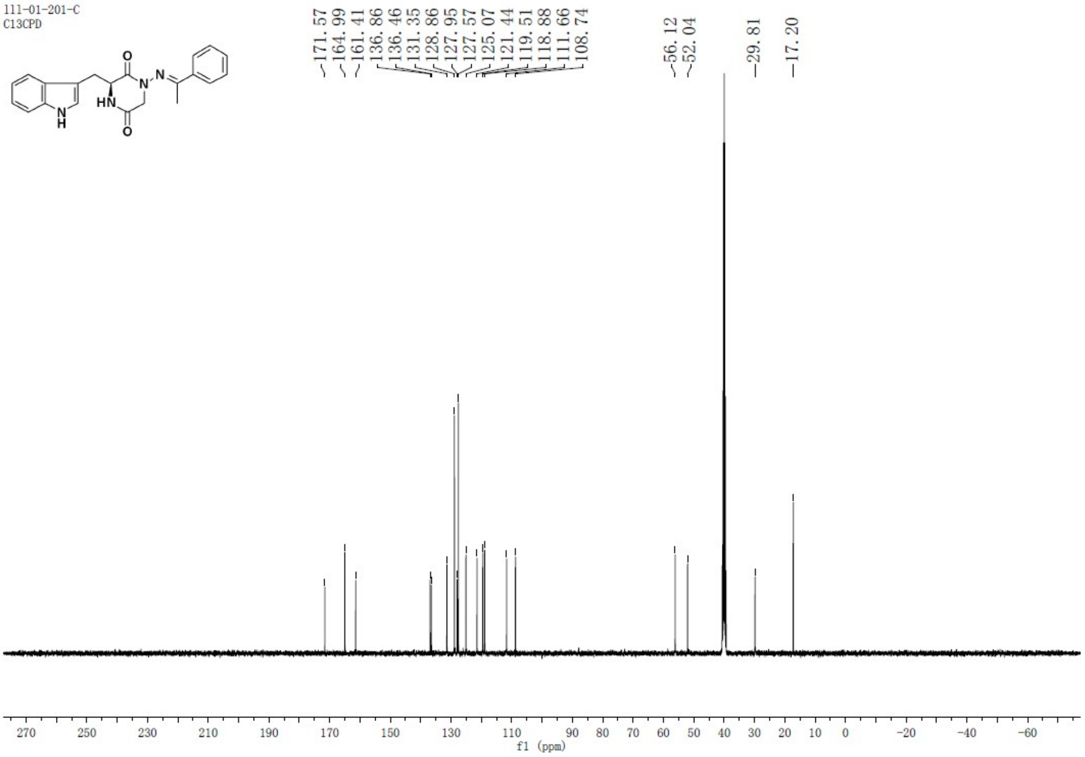


**Figure S50** $^{13}$C NMR (100 MHz, DMSO-$d_6$) of **25**

**26**

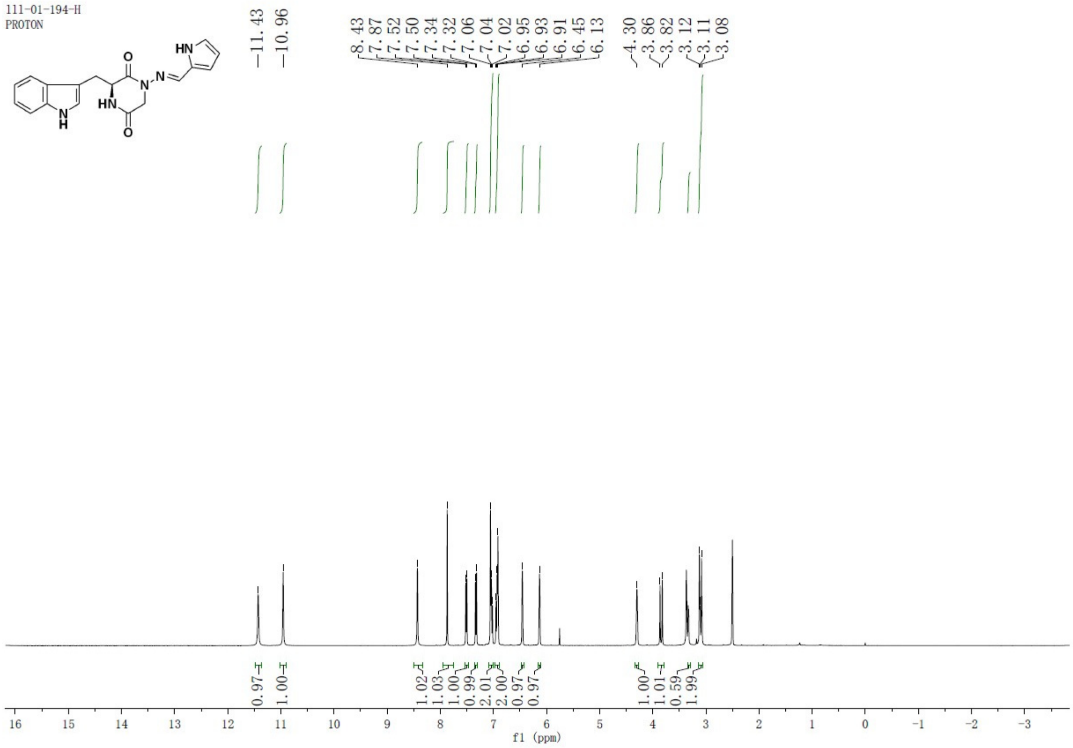


**Figure S51** $^{1}$H NMR (400 MHz, DMSO-$d_6$) of **26**

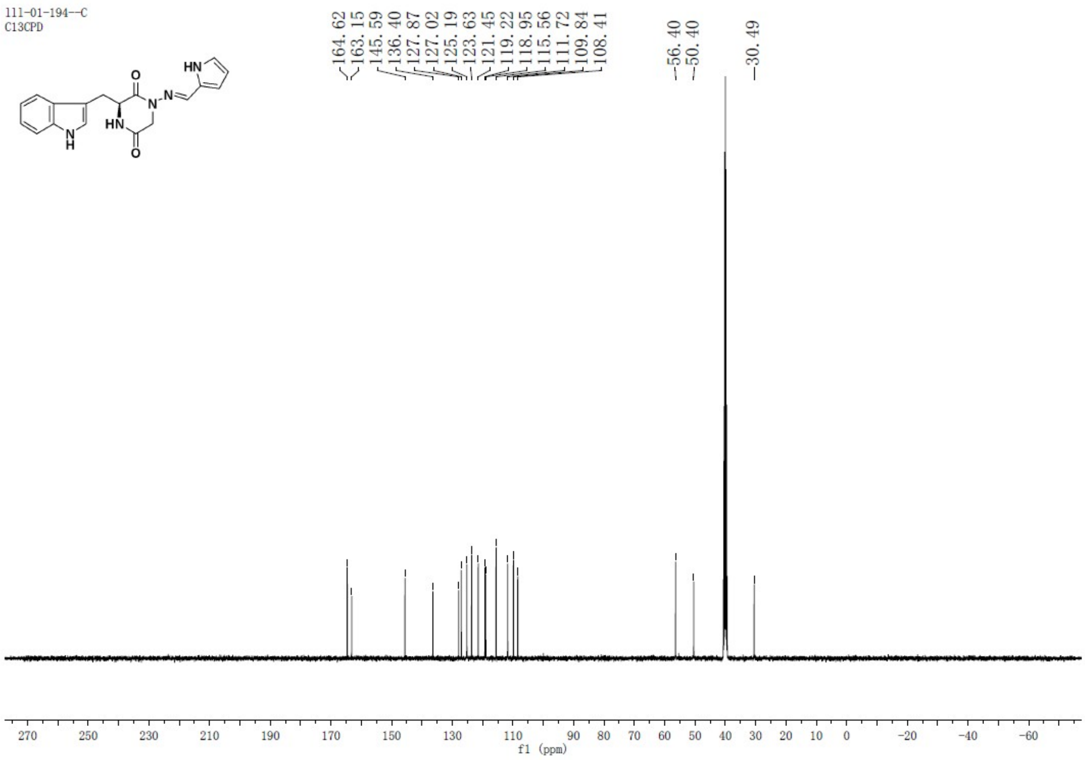


**Figure S52** $^{13}$C NMR (100 MHz, DMSO-$d_6$) of **26**

**27**

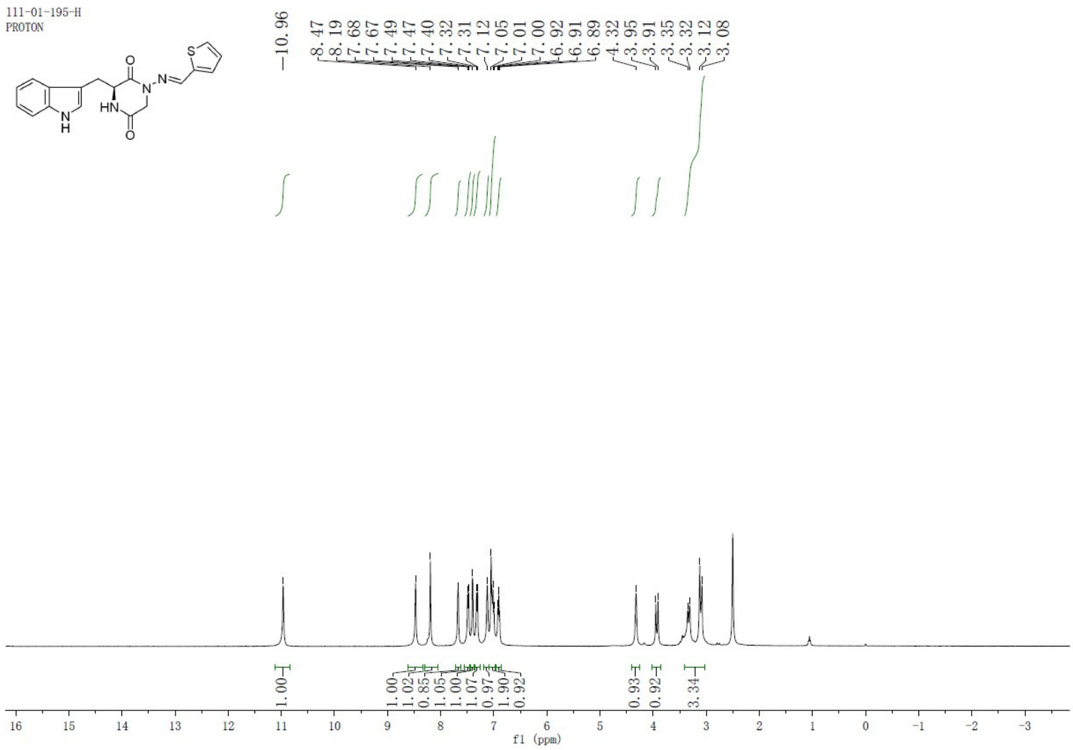


**Figure S53** $^{1}$H NMR (400 MHz, DMSO-$d_6$) of **27**

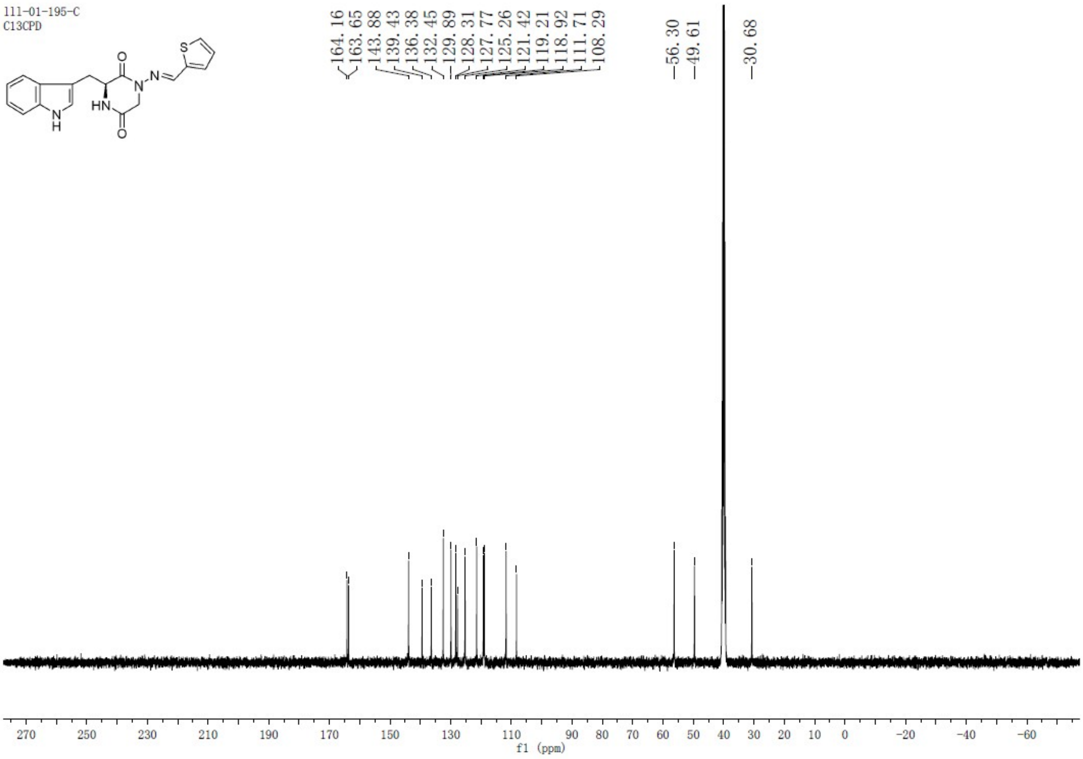


**Figure S54** $^{13}$C NMR (100 MHz, DMSO-$d_6$) of **27**

# 28

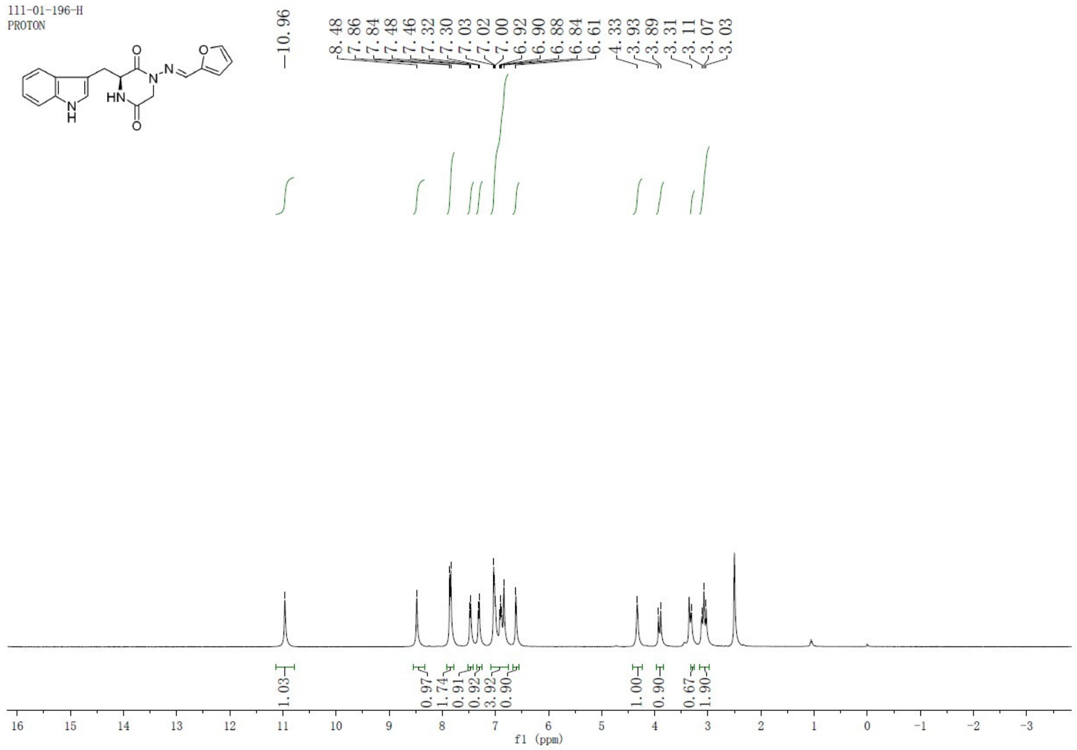


**Figure S55** $^{1}$H NMR (400 MHz, DMSO-$d_6$) of **28**

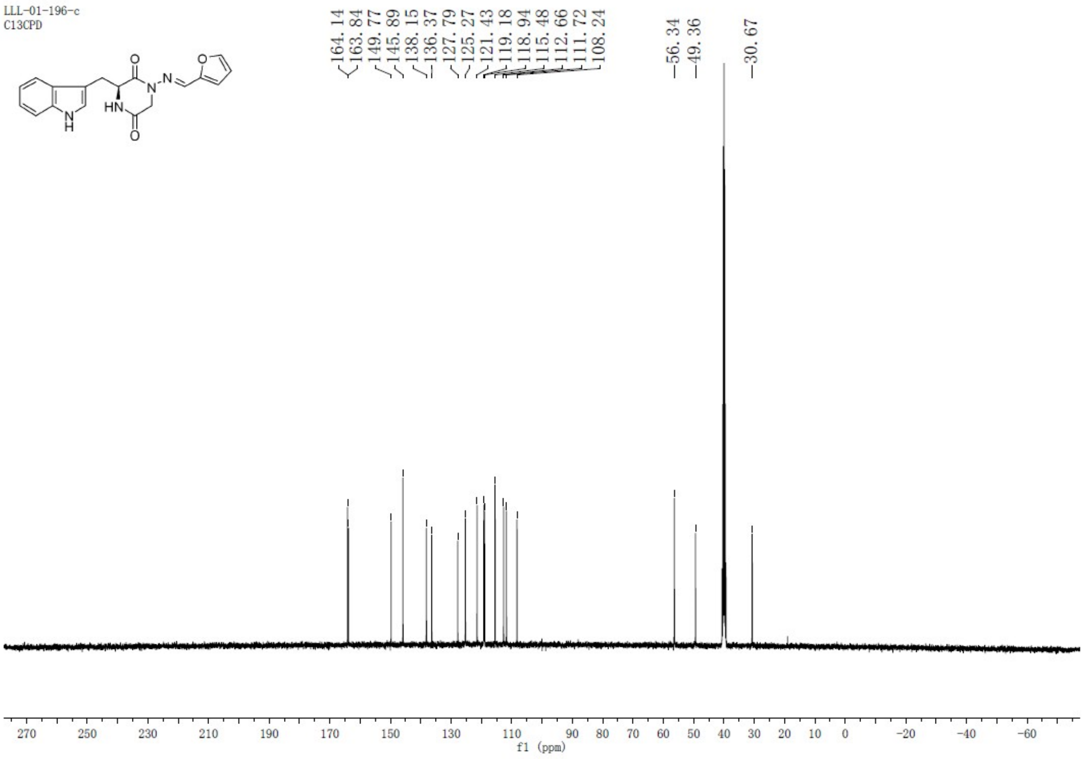


**Figure S56** $^{13}$C NMR (100 MHz, DMSO-$d_6$) of **28**

**29**

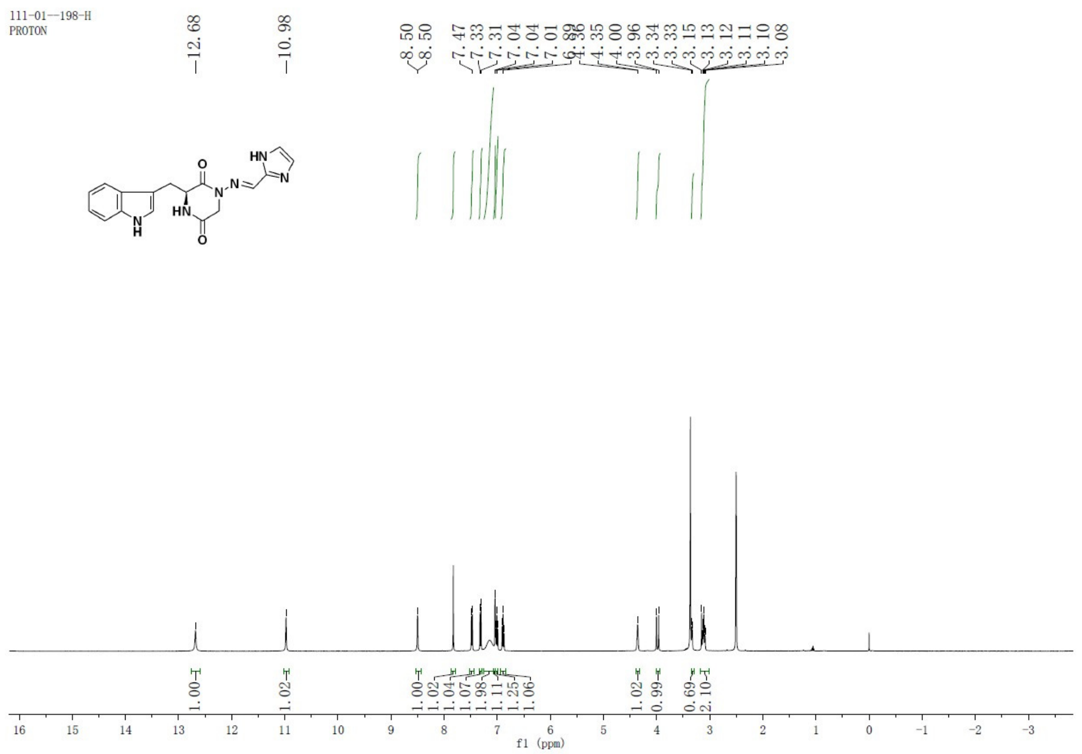


**Figure S57** $^{1}$H NMR (400 MHz, DMSO-$d_6$) of **29**

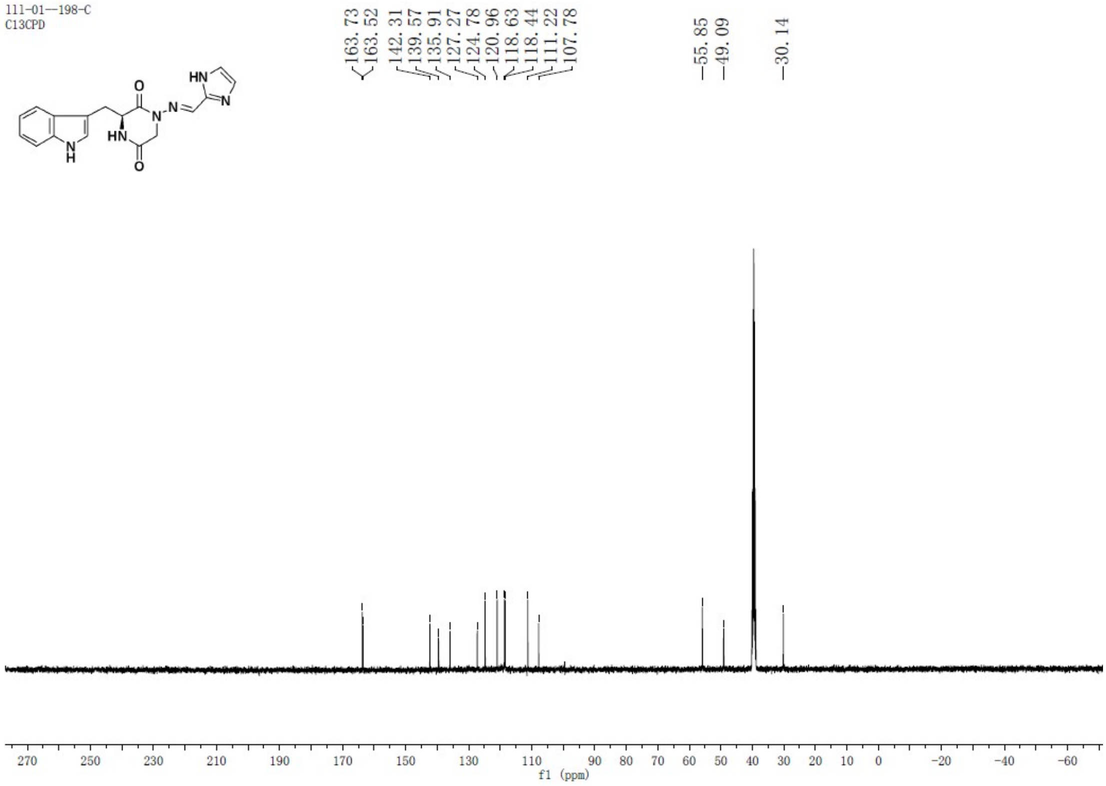


**Figure S58** $^{13}$C NMR (100 MHz, DMSO-$d_6$) of **29**

**30**

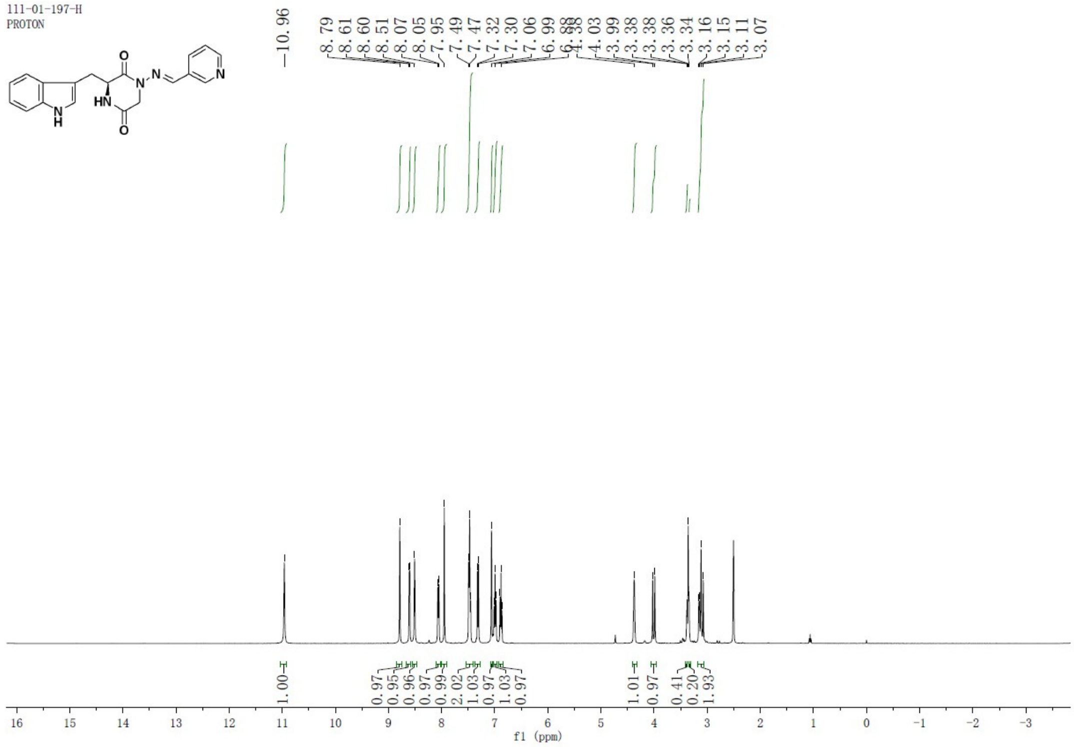


**Figure S59** $^{1}$H NMR (400 MHz, DMSO-*d*$_6$) of **30**

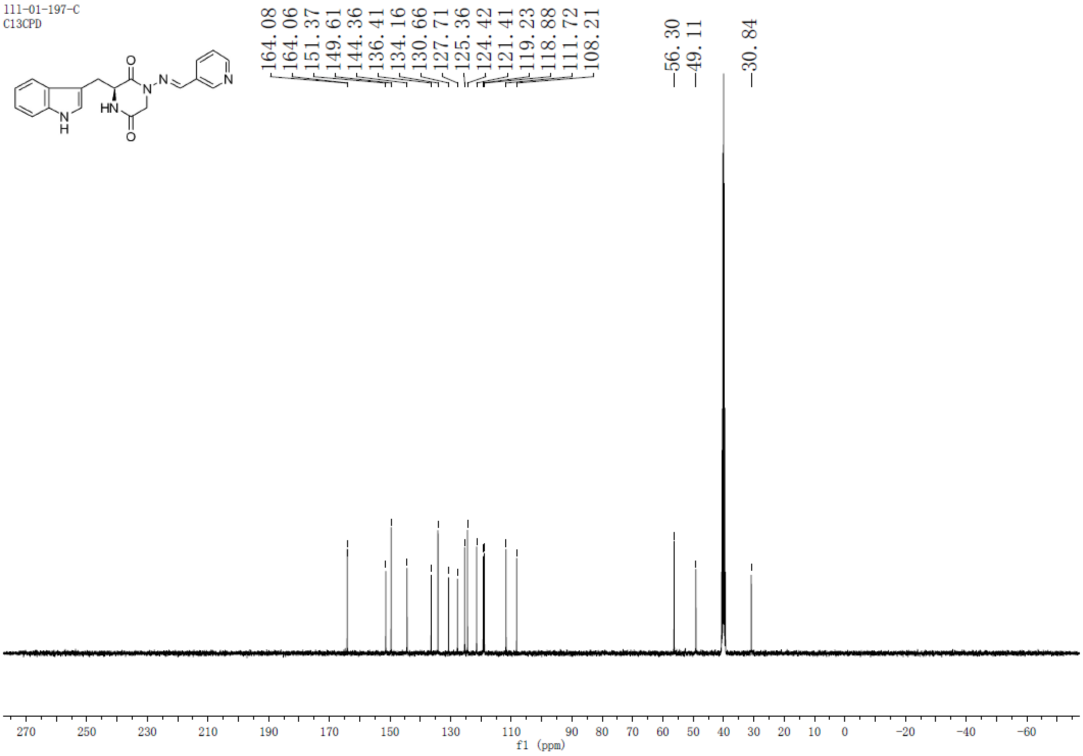


**Figure S60** $^{13}$C NMR (100 MHz, DMSO-*d*$_6$) of **30**

# 31

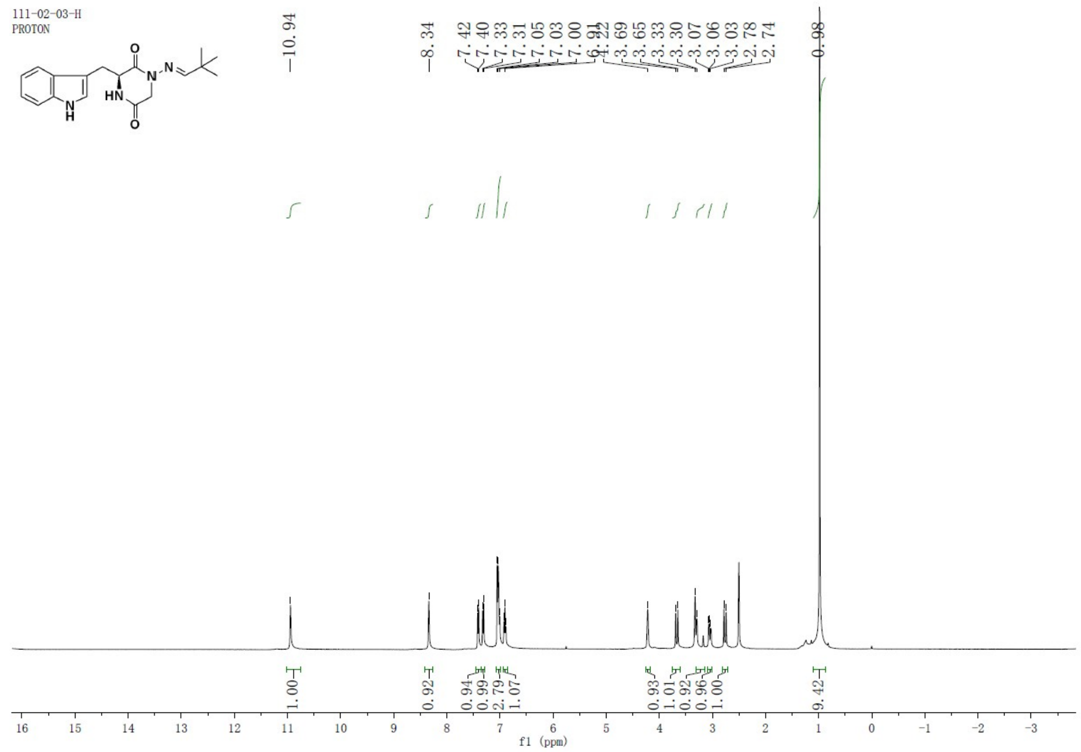


**Figure S61** $^{1}$H NMR (400 MHz, DMSO-$d_6$) of **31**

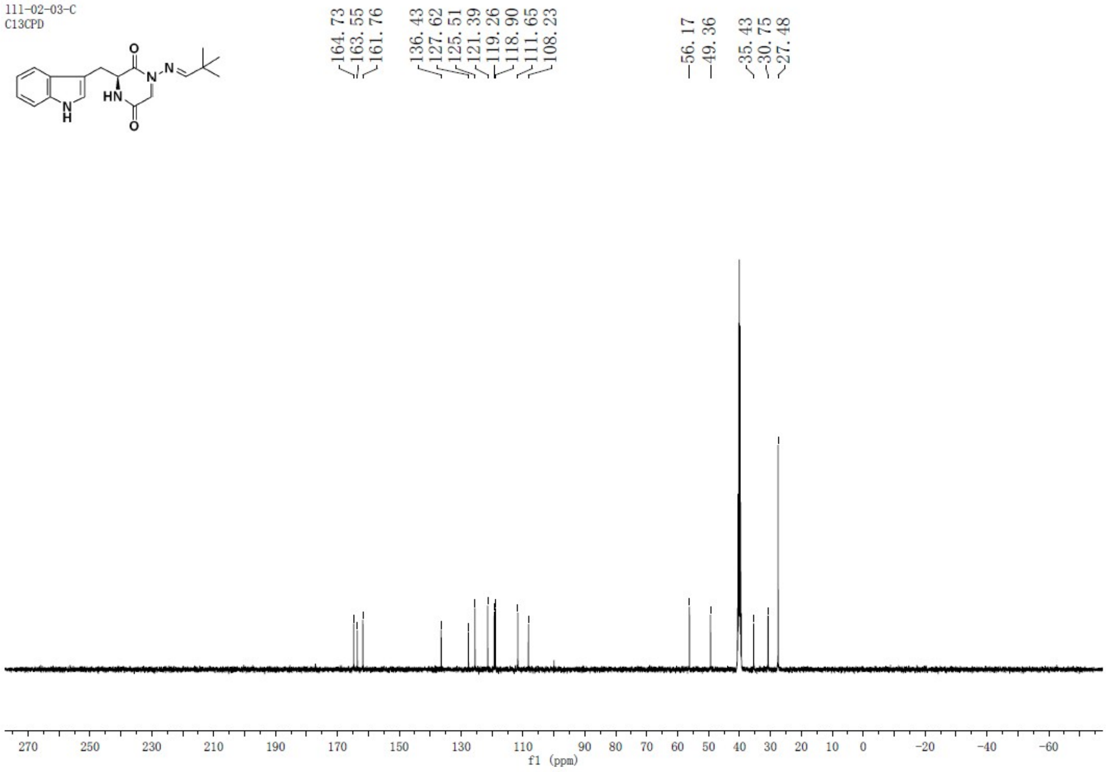


**Figure S62** $^{13}$C NMR (100 MHz, DMSO-$d_6$) of **31**

**32**

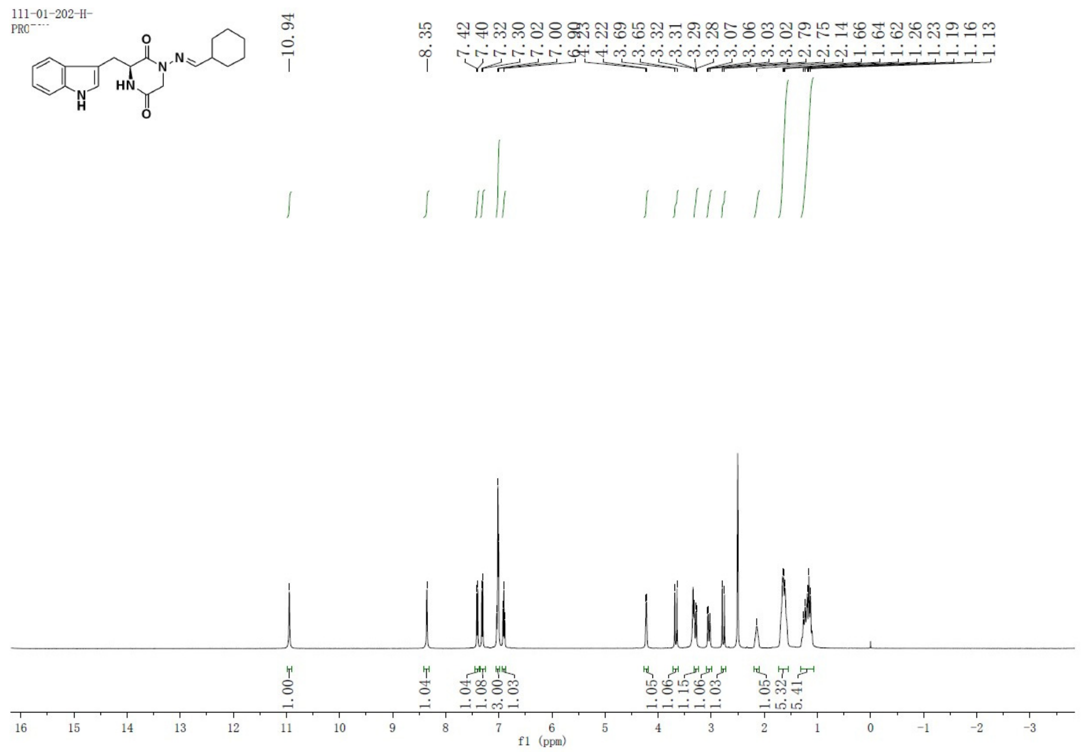

**Figure S63** $^1$H NMR (400 MHz, DMSO-$d_6$) of **32**

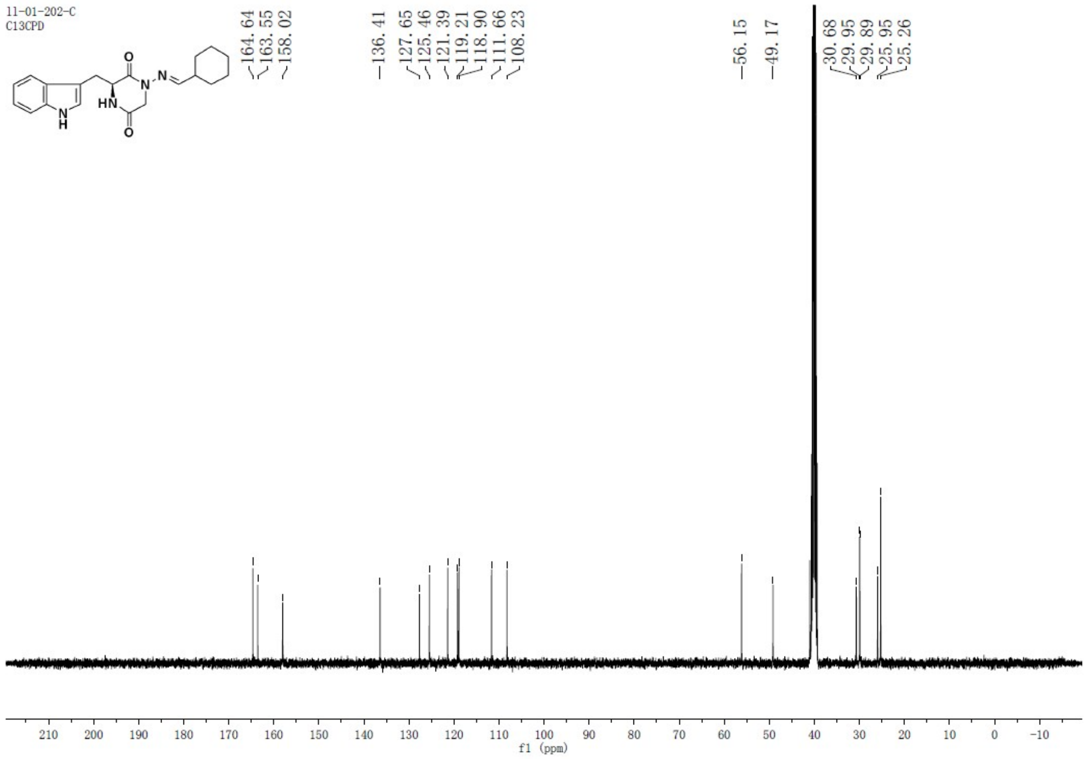

**Figure S64** $^{13}$C NMR (100 MHz, DMSO-$d_6$) of **32**

## Section S2: Detailed Bioassay Procedures for Anti-TMV Activities

**Compound solution preparation**: Test compound was dissolved within a suitable amount of *N*,*N*-dimethyl formate and diluted with water containing 0.1% TW-80 to make a concentration of 500μg/mL, and the aqueous solution was diluted to 100 μg/mL.

**Antiviral Activity of Compounds against TMV *in Vitro*.** Fresh leaf of the 5-6 growth stage of tobacco (*N. tabacum L.*) inoculated by the juice-leaf rubbing method (concentration of TMV was 6 × $10^{-3}$ μg/mL) was cut into halves along the main vein. The halves were immersed into the solution of test compounds and solvent (double-distilled water containing 0.1% TW-80) for 30 min, respectively, and then cultured at 25 °C for 72 h. The local lesion numbers were then counted. Each compound was replicated at least 3 times.

**Protective Effect of Compounds against TMV *in Vivo*.** The compound solution was smeared on the growing *N. tabacum L.* leaves of the same ages. Another pot was smeared with solvent for control. After 12 h, the leaves were inoculated by the juice-leaf rubbing method and then washed with water. The local lesion numbers appearing 3-4 days after inoculation were counted. There were three replicates for each compound.

**Inactivation Effect of Compounds against TMV *in Vivo***. The virus was inhibited by mixing with the compound solution at the same volume for 30 min. The mixture was then inoculated on the growing leaves of the same ages, whereas another pot was inoculated with the mixture of solvent and the virus for control. The local lesion numbers were recorded 3–4 days after inoculation. There were three replicates for each compound.

**Curative Effect of Compounds against TMV *in Vivo***. TMV (concentration of 6.0 × $10^{-3}$ mg/mL) was inoculated on the growing leaves of *N. tabacum L.* of the same ages. Then, the leaves were washed with water and dried. The compound solution was smeared on the leaves, whereas another pot was smeared with solvent for control. The local lesion numbers were then counted and recorded 3–4 days after inoculation. There were three replicates for each compound.

The *in vitro* and *in vivo* inhibition rates of the compound were then calculated according to the following formula ("av" means average, and controls were not treated with compound):

inhibition rate (%) = [(av local lesion number of control - av local lesion number of drugtreated)/av local lesion number of control] × 100%.

## Section S3: Detailed Bioassay Procedures for the Fungicidal Activities

The compounds were evaluated in mycelial growth tests in artificial media against 14 plant pathogens at rate of 50 mg $L^{-1}$.

Test compound was dissolved within a suitable amount of acetone and diluted with water containing 0.1% TW-80 to the concentration of 500 mg $L^{-1}$. To each petri dish was added 1 mL such solution and 9 mL culture medium to make a 50 mg $L^{-1}$ of medicated tablet, whereas to another petri dish was added 1 mL sterilized water and 9 mL culture medium as blank control. A diameter of 4 mm of hyphae was cut by a whole puncher along the hyphae for bacteria to the outer plate and moved to the medicated tablet. Each treatment was performed three times. The dishes were stored in controlled environment cabinets (24 ± 1 °C) for 48 h, after which the diameter of mycelia growth was investigated and percentage inhibition was calculated. There were three replicates for each compound.

Percentage inhibition (%) = (averaged diameter of mycelia in blank controls - averaged diameter of mycelia in medicated tablets) / averaged diameter of mycelia in blank controls.

## Section S4: Stomach Toxicity against Lepidopteran Pests

The stomach toxicities of the title compounds against *P. xylostella, M. separata*, *H. armigera*, and *P. nubilalis* were tested according to the leaf-dip method using the reported procedure. Leaf disks (about 5 cm) were cut from fresh corn leaves and then were dipped into the test solution for 3–5 s. After air drying, the treated leaf disks were placed individually into a glass-surface vessel (7 cm). Each dried treated leaf disk was infested with 10 third-instar *P. xylostella,* or *M. separata*, or *H. armigera*, or *P. nubilalis*. Percentage mortalities were evaluated 4 days after treatment. Leaves treated with acetone were provided as controls. Each treatment was performed three times.

## Section S5: Larvicidal Activities against *Culex Pipiens Pallens*.

10 fourth-instar mosquito larvae were put into the 10 mL of the test solution. Percentage mortalities were evaluated 8 days after treatment. Evaluations were based on a percentage scale of 0–100, where 0 equals no activity and 100 equals total kill. Each treatment was performed three times. Error of the experiments was about 5%.

## Section S6: Acaricidal Activity against Larvae of *Tetranychus Cinnabarinus*

Each test compound was dissolved in acetone, and the acetone solution was diluted with distilled water to the desired concentration (200–0.05 mg/L). Fresh sieva bean leaves infested with mite larvae (60–100 larvae per leaf) were dipped into the test solution and swirled around for 3 s and then placed in a tube (10 cm inner diameter) lined with a piece of filter paper. The bioactivities were evaluated 4 days after treatment and are reported as mortality percentages, where 0% indicates no activity and 100% indicates a total kill.
